# Catalytic role of formaldehyde in particulate matter formation

**DOI:** 10.1073/pnas.2113265119

**Published:** 2022-01-31

**Authors:** Eleni Dovrou, Kelvin H. Bates, Jonathan M. Moch, Loretta J. Mickley, Daniel J. Jacob, Frank N. Keutsch

**Affiliations:** ^a^John A. Paulson School of Engineering and Applied Sciences, Harvard University, Cambridge, MA 02138;; ^b^Department of Earth and Planetary Sciences, Harvard University, Cambridge, MA 02138;; ^c^Department of Chemistry and Chemical Biology, Harvard University, Cambridge, MA 02138

**Keywords:** formaldehyde, dissolved sulfur dioxide, catalysis, hydroxymethyl hydroperoxide, hydroxymethanesulfonate

## Abstract

Formaldehyde (HCHO), the simplest and most abundant carbonyl in the atmosphere, contributes to particulate matter (PM) formation via two in-cloud processing pathways. First, in a catalytic pathway, HCHO reacts with hydrogen peroxide (H_2_O_2_) to form hydroxymethyl hydroperoxide (HMHP), which rapidly oxidizes dissolved sulfur dioxide (SO_2,aq_) to sulfate, regenerating HCHO. Second, HCHO reacts with dissolved SO_2,aq_ to form hydroxymethanesulfonate (HMS), which upon oxidation with the hydroxyl radical (OH) forms sulfate and also reforms HCHO. Chemical transport model simulations using rate coefficients from laboratory studies of the reaction rate of HMHP with SO_2,aq_ show that the HMHP pathways reduce the SO_2_ lifetime by up to a factor of 2 and contribute up to ∼18% of global sulfate. This contribution rises to >50% in isoprene-dominated regions such as the Amazon. Combined with recent results on HMS, this work demonstrates that the one-carbon molecules HMHP and HCHO contribute significantly to global PM, with HCHO playing a crucial catalytic role.

## Significance

Particulate matter, often formed via cloud processing, strongly influences the Earth’s climate and air quality. Particle composition depends on anthropogenic and biogenic emissions. Thus, in order to understand climate change, knowledge of the difference between preindustrial and current conditions is critical. Under preindustrial conditions, multifunctional organic hydroperoxides, which are strong oxidants and have the ability to contribute to particulate matter formation, are in higher concentrations in the atmosphere. In this work, we focus on the previously unknown importance of hydroxymethyl hydroperoxide, which can be formed by gas-phase reactions and in-cloud reaction of hydrogen peroxide with the simplest aldehyde, formaldehyde, revealing the catalytic role of formaldehyde, and demonstrate that this chemistry is of great importance for particle formation.

Particulate matter (PM) formation has a significant impact on cloud properties, climate, and human health (1). Higher toxicity has been attributed to small-size particles (particles with aerodynamic diameter smaller than 2.5 μm) due to their ability to enter the respiratory and cardiovascular system, causing adverse health effects in highly polluted environments (2–7). A substantial fraction of PM corresponds to sulfate, which is primarily formed from atmospheric oxidation of SO_2_ emitted anthropogenically by coal combustion and smelters (8). Important SO_2_ oxidation pathways include reaction with the hydroxyl radical (OH) in the gas phase and reaction with hydrogen peroxide (H_2_O_2_), ozone, multifunctional organic hydroperoxides (RXOOH), and NO_2_ in cloud droplets (9–13). Simple organic hydroperoxides, such as methylhydroperoxide and peroxyacetic acid, are unlikely to contribute due to their low Henry’s law constants, on the order of 10^2^ to 10^3^ M ⋅ atm^−1^ and lower rate constants for oxidation of SO_2_, up to 10^2^ M^−1^ ⋅ s^−1^ compared to multifunctional organic hydroperoxides (RXOOH), such as isoprene hydroxyl hydroperoxides (ISOPOOH), which have two orders of magnitude higher Henry’s law constants and at least an order of magnitude higher oxidation rate constants (9, 12). The contribution of transition metal chemistry and reactions on the surface of aerosols are more uncertain but could also play an important role (14). Formation of sulfate by SO_2_ oxidation competes with removal of SO_2_ by deposition (15). A complete understanding of the contribution of all pathways is important to quantify the SO_2_ lifetime (τSO_2_) against oxidation and the implied formation rate of sulfate, especially as recent studies showed that models underestimate sulfate in SO_2_ source regions (16–23). Competition between gas-phase and condensed-phase pathways has a crucial role in new particle formation (9). Understanding sulfur oxidation pathways is especially important under changing atmospheric conditions (e.g., in areas where observed decreases in anthropogenic NO_x_ emissions are observed, such as in the southeast United States [SE-US]).

In-cloud oxidation of SO_2,aq_ to sulfate is considered the main source of global PM sulfur, with H_2_O_2_ presumed to be the most important oxidant (9–11, 24). Atmospheric models overestimate the global sulfur dioxide concentration by ∼50%, but some also underestimate the sulfate concentration by ∼20% globally and >20% on regional scales (16–23). Kim et al. (23) reported that GEOS-Chem underestimates sulfate by 34% in the SE-US. Similarly, Pai et al. (21) showed that GEOS-Chem underestimates sulfate significantly in low-NO_x_ isoprene-rich regions such as the Amazon and the SE-US. Simulations using large-scale sulfate aerosol models or a global chemical tracer model have shown overestimation of SO_2(g)_ and underestimation of sulfate in the Northern Hemisphere during winter (16, 17). Studies with Lagrangian atmospheric models across Europe and North America also underestimate sulfate in winter but overestimate sulfate during summer (19, 20). Modeling additional SO_2_ oxidation pathways in these regions could decrease τSO_2_ and increase regional sulfate production and burden.

Another important compound that could contribute to PM formation is formaldehyde. Formaldehyde (HCHO) is the most abundant and simplest carbonyl in the atmosphere, formed primarily via photochemical oxidation of volatile organic compounds, such as isoprene, its main biogenic precursor (25). Having a high Henry’s law constant ($∼5.5\cdot 10^{3}\;\text{M}\cdot {\text{atm}}^{-1}$) (26), HCHO can partition into cloud and fog water. The term HCHO, as used in this work, refers to both free HCHO and its hydrated form, which is the dominant form in clouds and fog droplets. In cloud droplets, HCHO reacts with SO_2,aq_ to form hydroxymethanesulfonate (HMS; ${\text{HOCH}}_{2}{\text{SO}}_{3}^{-}$) (27–30). HMS is stable at pH <6 and resistant to oxidation by hydrogen peroxide (H_2_O_2_) and ozone (O_3_) but can be oxidized by OH to form sulfate and reform HCHO in the aqueous phase (31–34). HMS has recently received attention primarily from the perspective of the PM sulfur budget (30, 35), whereas contribution to the organic carbon budget has not been explicitly discussed. HCHO also reacts with aqueous H_2_O_2_ to form hydroxymethyl hydroperoxide (HMHP) (36). HMHP is also formed in the gas phase via reaction of the CH_2_OO Criegee intermediate with water (36). HMHP has a higher Henry’s law constant ($∼2\cdot 10^{6}\;\text{M}\cdot {\text{atm}}^{-1}$) (37) than H_2_O_2_, and as an RXOOH, it has the potential to be an efficient oxidant for SO_2,aq_, as recently shown for ISOPOOH (12).

In this work, we evaluate the contribution of two species with only one carbon atom, HCHO and HMHP, to PM formation. Laboratory results are integrated into the GEOS-Chem model in order to investigate the importance of HCHO and HMHP to global and regional PM. We calculate the contribution of a pathway, the oxidation of SO_2,aq_ by HMHP, to PM formation. We discuss the role HCHO plays, especially in the competition between 1) formation of HMS via direct reaction of HCHO with SO_2,aq_ and 2) formation of sulfate via reaction of HCHO with H_2_O_2_ to form HMHP, which subsequently oxidizes SO_2,aq_ to sulfate and reforms HCHO.

## Results

### Equilibrium and Kinetics of Aqueous HCHO + H_2_O_2_ ⇌ HMHP.

The HMHP equilibrium has been investigated previously (36, 38, 39). In this work, NMR spectroscopy (^1^H-NMR) was used to determine the equilibrium constant and kinetics of HMHP formation from formaldehyde.

The equilibrium reaction is expressed as

$${\text{H}}_{2}{\text{O}}_{2}+\text{HCHO}\overset{k_{f}}{\underset{k_{r}}{⇌}}\text{HMHP.}$$

The equilibrium constant obtained was ${\text{K}}_{\text{eq}}=172(\pm 2){\;\text{M}}^{-1}$, in excellent agreement with the literature (*SI Appendix*, Table S1) (36, 38, 39).

We also conducted experiments to determine the forward and reverse rate constants over a range of pH values and compared with literature values (experimental procedure in *Materials and Methods*). The equilibrium constant is expressed as

[1] $${\text{K}}_{\text{eq}}=\frac{{\left[\text{HMHP}\right]}_{\text{eq}}}{{\left({\left[\text{HCHO}\right]}_{\text{o}}-{\left[\text{HMHP}\right]}_{\text{eq}}\right)}^{2}}$$

for our experimental conditions, which had equal initial concentrations of ${\left[{\text{H}}_{2}{\text{O}}_{2}\right]}_{\text{o}}$ and ${\left[\text{HCHO}\right]}_{\text{o}}$. ${\left[\text{HMHP}\right]}_{\text{eq}}$ is the HMHP equilibrium concentration, and

[2] $${\left[{\text{H}}_{2}{\text{O}}_{2}\right]}_{\text{eq}}={\left[\text{HCHO}\right]}_{\text{eq}}={\left[\text{HCHO}\right]}_{\text{o}}-{\left[\text{HMHP}\right]}_{\text{eq}}.$$

The rate of change in [HMHP] is

[3] $$\frac{d\left[\mathrm{HMHP}\right]}{\mathrm{dt}}=k_{f}\cdot {\left[\text{HCHO}\right]}^{2}-k_{r}\cdot [\text{HMHP}]$$

for our experimental conditions and at equilibrium.

By observing the increase in the HMHP signal intensity over time, the forward rate constant of HMHP, ${\text{k}}_{\text{f}}$, was experimentally determined with lower limit of ${\text{k}}_{\text{f},\text{meas}}\geq 1.3\;\left(\pm 0.8\right){\;\text{M}}^{-1}\cdot {\text{s}}^{-1}$, the value in agreement with the average forward rate constant reported in the literature (38, 39). The reverse rate constant, ${\text{k}}_{\text{r}}$, calculated from ${\text{K}}_{\text{eq}}=\frac{{\text{k}}_{\text{f},\text{meas}}}{{\text{k}}_{\text{r}}}$, is ${\text{k}}_{\text{r},\text{calc}}\geq 7.6\left(\pm 0.6\right)\;\cdot 10^{-3}{\;\text{s}}^{-1}$ (*Materials and Methods*). By observing the decrease of the HMHP signal intensity upon dilution, the reverse rate constant was measured to be ${\text{k}}_{\text{r},\text{meas}}=6\;\left(\pm 2\right)\cdot 10^{-7}{\;\text{s}}^{-1}$, and the forward rate constant calculated from this is ${\text{k}}_{\text{f},\text{calc}}=1.0\;\left(\pm 0.2\right)\cdot 10^{-4}{\;\text{M}}^{-1}\cdot {\text{s}}^{-1}$. The literature values for ${\text{k}}_{\text{f}}$ also vary by more than an order of magnitude between $9.4\cdot 10^{-1}$ and $7.5\cdot 10^{-2}{\;\text{M}}^{-1}\cdot {\text{s}}^{-1}$, with the higher value being in close agreement with the one determined here; for ${\text{k}}_{\text{r}}$, the literature values vary between $6.3\cdot 10^{-3}$ and $5.9\cdot 10^{-4}{\;\text{s}}^{-1}$ (*SI Appendix*, Table S1). The literature values were obtained by observing the decrease and increase of H_2_O_2_ using either equal concentrations of HCHO and H_2_O_2_ or excess of HCHO (*SI Appendix*, Supplementary Discussion 2), whereas, in our study, HCHO and HMHP were both directly measured (38, 39). We were not able to resolve the discrepancy between ${\text{k}}_{\text{f},\text{meas}}$ and ${\text{k}}_{\text{r},\text{meas}}$. For that reason, and as ${\text{k}}_{\text{f},\text{meas}}$ is only a lower limit, we used three scenarios that correspond to fast, medium, and slow equilibria, K_f_, K_m_, and K_s_, respectively: 1) K_f_: an upper-bound formation rate constant is considered equal to ${\text{k}}_{\text{f},\text{max}}=100\;\left(\pm 35\right){\;\text{M}}^{-1}\cdot {\text{s}}^{-1}$, with the reverse rate constant equal to ${\text{k}}_{\text{r},\text{max}}=0.6\;\left(\pm 0.2\right){\;\text{s}}^{-1}$; 2) K_m_: the lower limit measured ${\text{k}}_{\text{f},\text{meas}}=1.3\;\left(\pm 0.8\right){\;\text{M}}^{-1}\cdot {\text{s}}^{-1}$ and ${\text{k}}_{\text{r},\text{calc}}=7.6\;\left(\pm 0.6\right)\;\cdot 10^{-3}{\;\text{s}}^{-1}$; and 3) K_s_: the measured decomposition rate constant,${\;\text{k}}_{\text{f},\text{calc}}=1.0\;\left(\pm 0.2\right)\cdot 10^{-4}{\;\text{M}}^{-1}\cdot {\text{s}}^{-1}$ and ${\text{k}}_{\text{r},\text{meas}}=\;6\;\left(\pm 2\right)\cdot 10^{-7}{\;\text{s}}^{-1}$. The impact of the different rate constants on our conclusions is discussed later.

### Rate of Oxidation of SO_2,aq_ via HMHP.

**Fig. 1.**
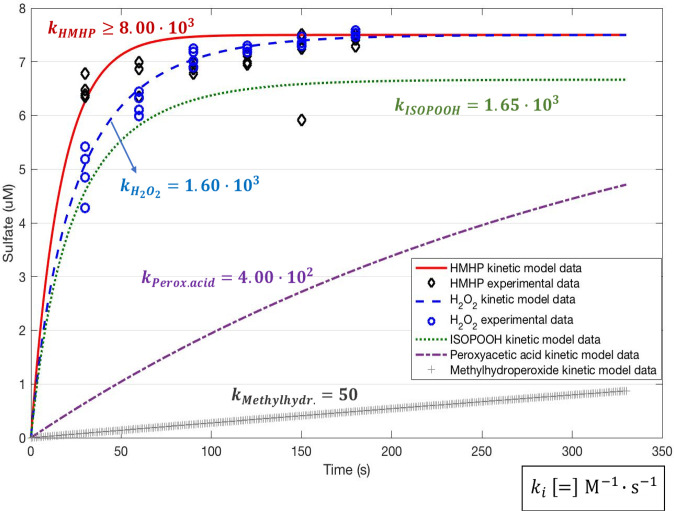
Sulfate production versus time due to oxidation of SO_2,aq_ via peroxides for pH = 5.5. The experimental data obtained in this work correspond to the sulfate production from the reaction of SO_2,aq_ with HMHP and H_2_O_2_. The formation rates of sulfate due to oxidation by ISOPOOH and the simple hydroperoxides, peroxyacetic acid and methylhydroperoxide, were simulated following the results of Dovrou et al. (12) and Lind et al. (9).

Experiments to determine the rate constant of oxidation of SO_2,aq_ by HMHP followed the protocol for recent work on the ISOPOOH and H_2_O_2_+ SO_2,aq_ reactions (*SI Appendix*, Table S2) (12). Sulfate formation was so rapid under experimental conditions (completion within 30 s; Fig. 1 and *SI Appendix*, Fig. S1) that only a lower limit for the rate constant was determined. Slower reaction rates could not be accessed due to the detection limit of the ion chromatography (IC). The lower limit of this rate constant, k(HMHP)$\;\geq 8.0(\pm 1.2)\cdot 10^{3}{\;\text{M}}^{-1}{\;\text{s}}^{-1}$ at pH = 5.5, shows that reaction of HMHP with SO_2,aq_ is significantly faster than reaction with H_2_O_2_ [k(H_2_O_2_)=$1.6\cdot 10^{3}{\;\text{M}}^{-1}{\;\text{s}}^{-1}$] or ISOPOOH [k(1,2-ISOPOOH)=$\;1.65\cdot 10^{3}{\;\text{M}}^{-1}{\;\text{s}}^{-1}$] (*SI Appendix*, Table S4) (9, 10, 12, 40–42). From experiments at lower pH of 4.5 and 3, we estimate a further increase in rate constants to $\geq 1.5(\pm 0.7)\cdot 10^{4}{\;\text{M}}^{-1}{\;\text{s}}^{-1}$ and $\geq 1.4(\pm 0.8)\cdot 10^{4}{\;\text{M}}^{-1}{\;\text{s}}^{-1}$, respectively, similar to H_2_O_2_ and in contrast to ISOPOOH.

### GEOS-Chem Model Results.

**Fig. 2.**
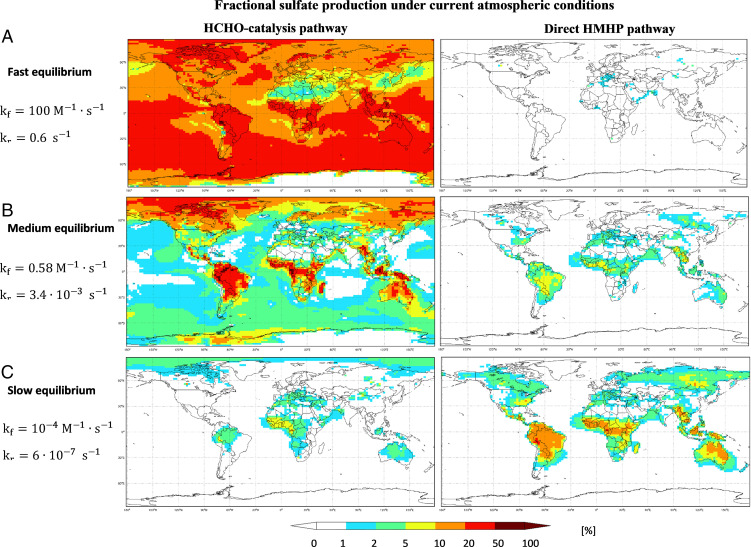
Contribution of the two HMHP pathways to column sulfate production. (*A*–*C*) The contribution is estimated for fast (*A*), medium (*B*), and slow (*C*) HMHP equilibrium under current atmospheric conditions from GEOS-Chem simulations at altitudes of 0 to 10 km. If the equilibrium is reached under the fast rate, the HCHO-catalysis pathways are dominant compared to the direct HMHP pathway for the oxidation of dissolved SO_2_.

**Fig. 3.**
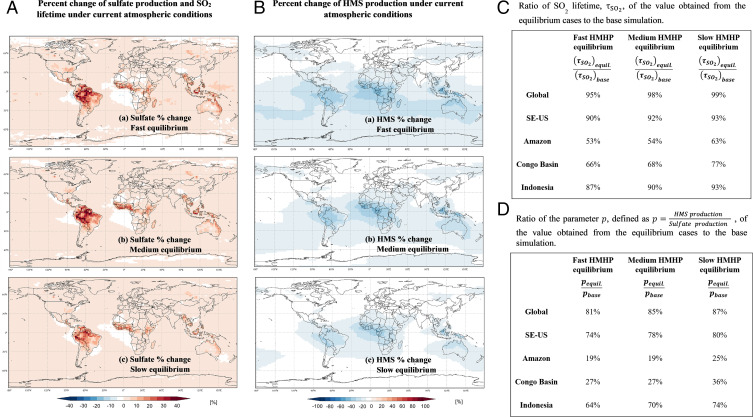
Percent change in column sulfate and HMS production and ratio of SO_2_ lifetime and HMS to sulfate production. (*A* and *B*) Percent change in column sulfate (*A*) and HMS (*B*) production considering fast, medium, and slow HMHP equilibrium under current atmospheric conditions at altitudes of 0 to 10 km. The GEOS-Chem model described in this work was used to perform the simulations. (*C*) The percentages presented in the table represent the ratio of SO_2_ lifetime, $\tau_{SO_{2}}$, of the value obtained from the equilibrium cases to that from the base simulation for the Amazon, SE-US, Congo Basin, and Indonesia. The percentages are all lower than 100%; thus, the equilibrium cases reduce the SO_2_ lifetime. (*D*) The percentages presented in the table represent the ratio of the parameter $\text{p}=\frac{\text{production}\;(\text{HMS})}{\text{production}\;({\text{SO}}_{4}^{2-})}$ of the value obtained from the equilibrium cases to that from the base simulation for the Amazon, SE-US, Congo Basin, and Indonesia. The percentages are all lower than 100%; thus, the equilibrium cases reduce the production of HMS compared to sulfate (*SI Appendix*, Tables S5–S7).

To examine the effect of HMHP/HCHO reactions on sulfate and HMS formation, GEOS-Chem simulations under current, C, and “preindustrial,” PI, atmospheric conditions were performed (*Materials and Methods*). Two HMHP pathways of sulfate formation were considered for the GEOS-Chem simulations: 1) HMHP formed in the gas-phase ozonolysis of terminal alkenes with subsequent partitioning into cloud water (HMHP-direct), where it decomposes to HCHO and H_2_O_2_, dependent on the equilibration rate, and 2) HMHP formed in the aqueous phase from the reaction of H_2_O_2_ with HCHO (HCHO-catalysis), also dependent on the rate of equilibration. Three HMHP equilibrium cases were considered in the GEOS-Chem simulations, all with K_eq_ = 172 ${\text{M}}^{-1}$: 1) fast equilibrium, K_f_, 2) medium equilibrium, K_m_, and 3) slow equilibrium, K_s_. We present the model contribution of all RXOOH (i.e., ISOPOOH, HMHP-direct and HCHO-catalysis) to sulfate formation (Fig. 2 and *SI Appendix*, Fig. S2) as well as to changes in the sulfate burden, τSO_2_, (Fig. 3 *A* and *C* and *SI Appendix*, Fig. S3 *A* and *C*) and the HMS/sulfate production ratio (Fig. 3 *B* and *D* and *SI Appendix*, Fig. S3 *B* and *D*).

The GEOS-Chem contribution of all RXOOH pathways to sulfate in the global troposphere for the K_f_ case is 18%/24% for C/PI. For the K_m_ case, the corresponding contributions are 7%/8%, and for K_s_, this is reduced to 4%/3%. For K_f_, H_2_O_2_ remains the most important oxidant globally but HCHO-catalysis is significant, contributing the majority of RXOOH pathways with 17%/23% of C/PI of sulfate (Fig. 2 and *SI Appendix*, Figs. S2 and S5). The contribution of HCHO-catalysis to global sulfate decreases as the equilibrium slows down to 5%/7% C/PI for the K_m_ case and 0.4%/0.6% for K_s_. The contribution of the HMHP-direct pathway increases but never exceeds 2.2% contribution to sulfate for the global troposphere, and H_2_O_2_ remains the dominant oxidant (*SI Appendix*, Tables S5–S7).

Regionally, RXOOH pathways contribute more to sulfate formation than other oxidation pathways (i.e., H_2_O_2_ and O_3_) in isoprene-rich regions (*SI Appendix*, Figs. S4 and S5), where they are dominant. Field measurements in the Amazon region have shown that local SO_2_ conversion to sulfate is important for PM formation 70% of the time (43). For K_f_, RXOOH pathways account for 79%/72% C/PI of sulfate formation in the lowest kilometer of the troposphere in the Amazon, 62%/52% in the Congo Basin, and 20%/58% in the SE-US. For K_f_, HCHO-catalysis contributes more than H_2_O_2_ itself in the Amazon and Congo Basin. The contribution of ISOPOOH is also significant in isoprene-rich regions (e.g., 20%/11% C/PI in the Amazon), adding to the importance of RXOOH for sulfate formation. In eastern China, which has higher NO_x_ than the Amazon, Congo, and SE-US, the current contribution of RXOOH is negligible for K_f_, but in the PI scenario, the RXOOH contribution rises to 40% nearly entirely from HCHO-catalysis. The HMHP-direct contribution stays below 1% for K_f_ (below 10% for K_s_), in part due to limited isoprene emissions in this region, while the contribution of gas-phase OH decreases by a factor of 5 and the contribution of H_2_O_2_ more than doubles to 43% for K_f_.

Similar to the global scale, a shift from HCHO-catalysis to HMHP-direct is also observed regionally with slower equilibria. However, while on the global scale, this shift is accompanied by a reduction in the contribution of RXOOH to sulfate formation by about a factor of 5, the corresponding contribution of RXOOH decreases only by an average of approximately one-fifth (19/23% C/PI) in the Amazon, one-third (30% in both C and PI) in the Congo Basin, and one-half (43%/60% C/PI) in the SE-US (*SI Appendix*, Tables S5–S7). For K_f_, HCHO-catalysis represents the most important RXOOH pathway in the Amazon while HMHP-direct does not contribute significantly. For K_s_, the contribution of HMHP-direct increases further and HCHO-catalysis is greatly reduced. Similar trends are observed for other regions with high isoprene emissions. Thus, due to partial compensation between the HMHP-direct and HCHO-catalysis pathways, the sum of the two contributions remains high in isoprene-rich environments even across the entire range of used HMHP equilibration rates (Fig. 2 and *SI Appendix*, Fig. S2).

While the global sulfate burden remains largely unchanged across the simulations, the large contribution of RXOOH pathways to sulfate formation in the Amazon, Congo Basin, and SE-US represents not simply a shift in which pathways form sulfate but also an increase in the regional sulfate burdens (e.g., 24%/22% on average in the Amazon for K_f_). These regional increases in sulfate burden are reflected in corresponding reductions of τSO_2_ (Fig. 3 *A* and *C* and *SI Appendix*, Fig. S3 *A* and *C* and Tables S8–S10). For the global troposphere, τSO_2_ is reduced only by 5%/7% C/PI, but in regions such as the Amazon and Congo Basin, τSO_2_ is roughly halved, a large change that decreases the already low gas-phase SO_2_ concentrations even further, especially under PI conditions. The increase in the regional sulfate burden is due to the increase in the local oxidation of SO_2,aq_, which reveals that the oxidation is outcompeting the sum of SO_2_ transport and deposition.

The RXOOH pathways also have significant impacts on the contribution of HMS to PM. The global HMS/sulfate production ratio is reduced by 19%/44% C/PI for the K_f_ case, 15%/44% for the K_m_ case, and 13%/38% for the K_s_ case (Fig. 3 *B* and *D* and *SI Appendix*, Fig. S3 *B* and *D* and Tables S5–S7). The reductions on regional scales can be much larger; for example, in the Amazon, the HMS/sulfate production ratio is reduced by 75 to 81% over all equilibrium cases. The same trend in impact on HMS/sulfate production ratio is observed in the Congo Basin and SE-US (Fig. 3 *B* and *D* and *SI Appendix*, Fig. S3 *B* and *D* and Tables S5–S7).

## Discussion

**Fig. 4.**
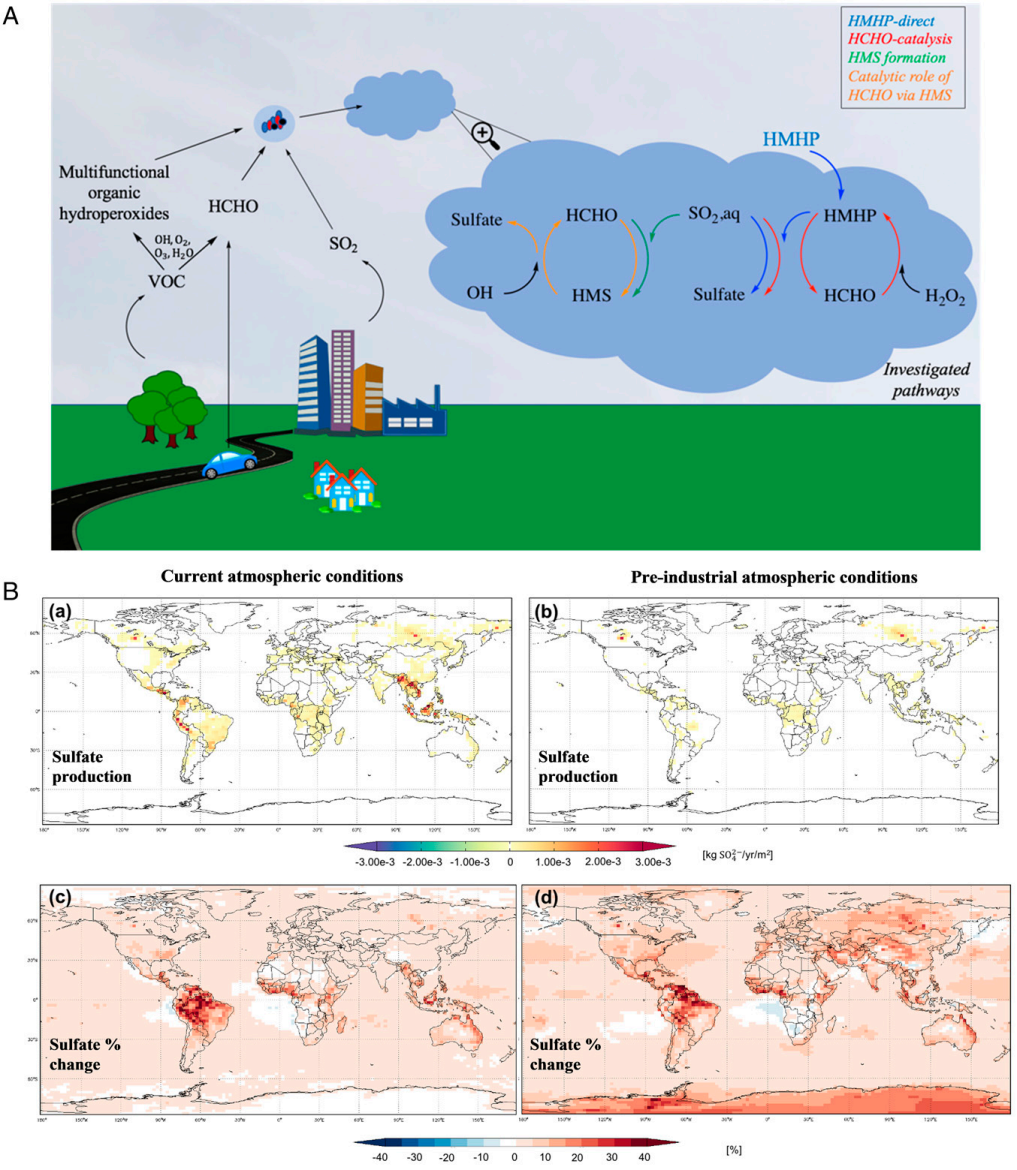
Schematic representation of the chemical processes investigated in this work and their effect on sulfate production and percent change under current and preindustrial atmospheric conditions. (*A*) The particular pathways of sulfur oxidation in cloud water discussed in this work are highlighted: oxidation by HMHP via two different routes (HMHP-direct and HCHO-catalysis) and HMS formation, including the catalytic role of HCHO. (*B*) Change in sulfate production (*a* and *b*) and percent change of sulfate production from base case to fast HMHP equilibrium case (*c* and *d*) under current (*a* and *c*) and preindustrial (*b* and *d*) atmospheric conditions. The fast HMHP equilibrium case is presented using the GEOS-Chem model described in this work. The values presented are annual averages at altitudes of 0 to 10 km.

Within cloud droplets and fog water, there exist competing pathways for HMHP (reaction with SO_2,aq_ versus dissociation to H_2_O_2_ and HCHO), H_2_O_2_ (reaction with SO_2,aq_ versus HCHO), HCHO (reaction with SO_2,aq_ versus H_2_O_2_), and SO_2,aq_ (reaction with H_2_O_2_ versus HMHP versus HCHO). The latter, SO_2,aq_ (reaction with H_2_O_2_ versus HMHP versus HCHO), is the most important to explain model results (Fig. 4*A* and *SI Appendix*, Fig. S6). Although the equilibrium constant for HMHP is large, the majority of HCHO and H_2_O_2_ remain in the free, or, for HCHO, hydrated, form under cloud droplet conditions. The fact that inclusion of the HMHP-direct and HCHO-catalysis pathways in the model greatly reduces the HMS/sulfate production ratio reflects not a direct competition between H_2_O_2_ and SO_2,aq_ for HCHO but, rather, that the oxidation via HCHO-catalysis or HMHP-direct is so rapid that less HMS is formed.

The lifetime of HMHP with respect to dissociation to HCHO and H_2_O_2_ increases from 1.7 s for K_f_ to 5 min for K_m_ and, finally, to 19 d for K_s_. For K_f_, the HMHP that partitions from the gas phase to the aqueous phase can be assumed to be instantaneously in equilibrium with HCHO and H_2_O_2_. In contrast, for K_s_, the dissociation of HMHP in the aqueous phase from the gas phase is so slow that it is irrelevant, whereas oxidation of SO_2,aq_ forming HCHO is an important pathway. This explains the shift of the contribution from HCHO-catalysis to HMHP-direct with slower equilibrium observed for the Amazon. For K_s_, H_2_O_2_ and HMHP are effectively not chemically coupled, and both can oxidize SO_2,aq_. In locations with high-HMHP mixing ratios (*SI Appendix*, Fig. S7), HMHP thus becomes an important oxidant due to the much higher rate constant (*SI Appendix*, Table S4) for K_s_ than H_2_O_2_. For K_f_, H_2_O_2_ and HMHP are coupled due to the fast equilibrium. In this case, the HCHO-catalysis pathway is very efficient as, despite low concentrations of HMHP, the rate constant is very high and HMHP is “instantaneously” regenerated from reformed HCHO and H_2_O_2_, which can be replenished from the gas phase. Although HMHP concentrations cannot be higher than those of H_2_O_2_, the HMHP turnover rate can be very high. This pathway is particularly effective, as HCHO is not consumed but regenerated. For medium equilibrium for ${\text{k}}_{\text{f}}=1.3{\;\;\text{M}}^{-1}\cdot {\text{s}}^{-1}$ and the literature lower limit ${\text{k}}_{\text{f},\text{literature}}=7.5\cdot 10^{-2}{\;\text{M}}^{-1}\cdot {\text{s}}^{-1}$, the estimated kinetic chain length is between 24 and 1.5 (*Materials and Methods*). This unexpected finding reveals the truly catalytic nature of this pathway, in which the oxidant can be viewed as H_2_O_2_, but with addition of HCHO as the catalyst.

The largest uncertainty in this work is the HMHP equilibration rate. Zhou and Lee showed that the equilibration rate is inversely dependent on [H^+^], increasing with higher pH (base catalysis) (36, 39). However, a critical finding is that regardless of this equilibration rate, HCHO-catalysis and/or HMHP-direct pathways are significant contributors to sulfate formation. This results in increased sulfate burdens in regions such as the Amazon and Congo Basin, accompanied by a reduction of τSO_2_ and HMHP (Fig. 3 and *SI Appendix*, Fig. S3 and Tables S8–S10). The effect is largest for the K_f_ case, but it is only slightly reduced for the K_s_ case, as the decrease in the contribution of HCHO-catalysis is largely balanced by increasing contribution of HMHP-direct. The reduction in τSO_2_ results in reductions in gas-phase SO_2_, in particular in regions that are already low in SO_2_, which acts as a limiting reagent in sulfate production so that H_2_O_2_ or HCHO concentrations are not affected. The reduced gas-phase SO_2_ in turn reduces production and concentration of gas-phase sulfuric acid. Analysis of the impact on new particle formation (NPF) is beyond the scope of this work, but, as formation of gas-phase sulfuric acid is critical for NPF, we expect this to have a significant impact on regional NPF.

The effects of PI versus C are more pronounced in regions that currently have high anthropogenic emissions, showing that the role of RXOOH could increase substantially as NO_x_ emissions decrease in such regions. In the SE-US, the contribution of HCHO-catalysis/HMHP-direct for the PI scenario is about three times that of C for the lowest km (*SI Appendix*, Tables S5–S7). Similar increases are revealed via the model simulations for the ISOPOOH contribution. For the C and PI scenario, inclusion of the RXOOH pathways reduces the HMS particulate sulfur production fraction by 20 to 26% and 85 to 88% compared to the base C/PI case, depending on the HMHP equilibration rate assumed. For India and East China, the contribution of RXOOH increases by factors of 5 and 25, respectively, due to the large changes in the NO_x_ regime for these regions. The Amazon and Congo Basin are low-NO_x_ environments regardless of C and PI scenarios, and there is little change in the contribution of HMHP/HCHO. The global contribution of the one-carbon pathways increases slightly in the PI compared to the C scenario, (Fig. 4*B* and *SI Appendix*, Fig. S8). In summary, the changes between C and PI are driven by changes in both the yields of RXOOH, as in East China, but also by changes in model OH production at the surface.

It is likely that other α-hydroxyhyperoxides, formed from highly water soluble, electron-poor aldehydes, such as hydroxy-acetone, glyoxal, or butenedial, react similarly to HMHP. It also has been shown that aldehyde-S(IV) species, such as glyoxal-S(IV), hydroxyacetaldehyde-S(IV), and the methylglyoxal adduct, are formed in cloud water and have faster formation rates than HMS at pH ≥4.5 (31). Although the solubility of these aldehydes is comparable to that of HCHO, their concentrations in the atmosphere are much lower (*SI Appendix*, Supplementary Discussion 1) (30, 34, 44–49). Nonetheless, studies are clearly needed to quantify their contribution and that of other multifunctional hydroperoxides, as only HMHP, an α-hydroxyhydroperoxide, and ISOPOOH, a β-hydroxyhydroperoxide, have been investigated, to our knowledge. Although these two likely are the most abundant multifunctional hydroperoxides, the sum of all multifunctional hydroperoxides could increase the importance of RXOOH pathways for the PM sulfur burden.

The HCHO-catalysis pathway reveals the importance of HCHO in cloud chemistry and sulfur PM formation. The additional HCHO pathway in cloud water, which results in HMS formation, contributes to organic PM formation, as HMS is an S(IV) organic compound. HMS decomposition is favored at pH values of approximately ≥5, which are observed in regions with dust and agricultural activities (50), resulting in the regeneration of HCHO upon reaction with OH (30, 34), which suggests a possible additional catalytic pathway of HCHO at pH ≥5. Therefore, this pathway could provide a perspective on HMS, in which HCHO, in this case, as well, acts as a catalyst for PM production.

This analysis clearly demonstrates that regardless of the rate of HMHP equilibration, one-carbon molecules HCHO and HMHP can have a significant impact on PM formation in the condensed phase via formation of sulfate and HMS. The discrepancy between the experimentally determined forward and reverse rates could possibly be addressed by online analysis tracking the peroxide concentration directly over a wider pH range. We show that HCHO plays an important role in formation of PM, acting as a catalyst. The oxidative environment can greatly influence the contribution of HMS versus sulfate, as HMHP formation is favored in the presence of H_2_O_2_ and HCHO and can rapidly result in sulfate formation. Lastly, as shown in previous studies, HMS can contribute significantly to the sulfur and organic PM budget, especially under haze conditions (e.g., in the northern Chinese winter haze conditions, HMS can reach up to 20 μg ⋅ m^−3^) (51). In such cases, the organic carbon PM contribution from the one-carbon molecule HCHO, via HMS, is estimated to be 2.2 μg ⋅ m^−3^, in accordance with the analysis in this work. In these conditions, a single chemical pathway of HCHO results in likely the most abundant single molecule, HMS, contributing to the PM carbon budget.

Comparing the GEOS-Chem simulations of this work with atmospheric models, it is observed that the implemented HMHP and ISOPOOH chemistry eliminates the underprediction of sulfate in some cases. Specifically, atmospheric models underpredict sulfate concentrations by ∼20% globally and >20% regionally (i.e., in the SE-USA by ∼34%) (16–23). In the case of fast equilibrium, the pathways account for 18% of sulfate globally and 58% in the SE-USA, contributions that can explain the global underpredicted sulfate and reveal a change in the contribution of the oxidative pathways to sulfate formation. For the cases of medium and slow HMHP equilibrium, the pathways account for 7% globally and 44% in the SE-US and 3% globally and 24% in the SE-USA, respectively. In both cases, the pathways’ contribution to sulfate formation in the SE-USA can explain the underpredicted values from previous model simulations. However, on a global scale, they contribute ∼1/6 to 1/3 of the underpredicted sulfate values.

The contribution of HCHO to PM production via HMS and HMHP, regardless of the rate of equilibration, impacts air quality and the Earth’s radiative balance as well as the acidity of clouds (Fig. 4*A* and *SI Appendix*, Fig. S9). The simplest aldehyde present in the atmosphere, HCHO, via its contribution to PM sulfur and carbon, affects, in an unexpected way, both climate and human health.

## Materials and Methods

### Chemicals, Sample Preparation, and Sample Analysis for the Oxidation Reaction of Sulfur Dioxide with the Examined Peroxides in the Aqueous Phase.

Sodium metabisulfite was purchased from Sigma-Aldrich (purity ≥99%) and used as the source of bisulfite (${\text{HSO}}_{3}^{-}$) in the solutions. HMHP was synthesized in the laboratory following the method of Zhao et al. (35). ${\text{H}}_{2}{\text{O}}_{2}$ was purchased from Sigma-Aldrich (30 wt. % in ${\text{H}}_{2}\text{O}$), and filtered Milli-Q water was used as a solvent. Each sample was prepared by using bisulfite and the examined peroxide. Separate experiments were conducted with ${\text{H}}_{2}{\text{O}}_{2}$ and HMHP as the dominant oxidant. For each examined reaction time, a new sample containing ${\text{HSO}}_{3}^{-}$ and peroxide and a control sample containing only ${\text{HSO}}_{3}^{-}$ were prepared and left to react. The production of sulfate (${\text{SO}}_{4}^{2-}$) due to oxidation by dissolved oxygen (${\text{O}}_{2}$) was monitored via the control samples and was subtracted from the results. Specifically, for each examined time, a control sample was prepared and analyzed prior to the analysis of the sample containing ${\text{HSO}}_{3}^{-}$ and a peroxide. The amount of ${\text{SO}}_{4}^{2-}$ formed in the control sample was calculated and subtracted from the amount of ${\text{SO}}_{4}^{2-}$ formed in the sample of ${\text{HSO}}_{3}^{-}$ with peroxide. The samples were analyzed at 25 °C over a pH range of 3 to 6. Reactant concentrations were in the micromolar range with reaction times of 0.5 to 3 min. The reactions were rapid relative to the sample analysis time; therefore, catalase (purchased from Sigma-Aldrich, catalase from bovine liver 2,000 to 50,000 units/mg protein) was used to quench the reaction after a given reaction time before the chromatographic analysis (12, 52). Hydrochloric acid (purchased from Sigma-Aldrich) was used to control sample pH.

A Dionex ICS-5000+ IC system was used to analyze the samples. The AG12A guard column and the AS12A analytical column (Dionex Ionpac) were selected in order to separate sulfur-containing species (34) and quantify sulfate production. The mobile phase during the experiments was 4.5 mM:1.4 mM sodium carbonate:sodium bicarbonate with flow rate 1.5 $\text{mL}\cdot {\text{min}}^{-1}$. The IC analysis was 10 min, as sulfate had a retention time of 7.5 min under the selected technical conditions of the analysis.

To examine the oxidation of dissolved SO_2_ by HMHP over a pH range of 3 to 6 (for which ${\text{HSO}}_{3}^{-}$ is the dominant form of dissolved SO_2_), the same experimental parameters presented by Dovrou et al. (12) were used. This provided a direct comparison of oxidation processes via ${\text{H}}_{2}{\text{O}}_{2}$, the ISOPOOH isomers, and HMHP. However, for HMHP, the results showed extremely rapid sulfate formation. Even with halved concentrations of ${\text{HSO}}_{3}^{-}$, ${\text{H}}_{2}{\text{O}}_{2}$ and HMHP sulfate formation was too fast to allow quantification, unlike ${\text{H}}_{2}{\text{O}}_{2}$ (*SI Appendix*, Table S2). Further reduction of concentrations was not feasible due to the detection limit of the IC system. All experiments were repeated four times, and each sample was prepared separately prior to the analysis. Temperature and pH were monitored and controlled during the reactions.

### HMHP Synthesis and Measurement of HMHP Equilibrium Constant.

The method described by Zhao et al. (36) was used to synthesize HMHP. Formaldehyde solution (37 wt. % in ${\text{H}}_{2}\text{O}$, purchased from Sigma-Aldrich) was mixed with hydrogen peroxide and left to react for at least 2 h under dark conditions at 25 °C. The concentrations of formaldehyde and hydrogen peroxide were $\frac{\left[\text{HCHO}\right]}{\left[{\text{H}}_{2}{\text{O}}_{2}\right]}=1$, and the mixture was analyzed using ^1^H-NMR to verify the formation of HMHP. Four sets of HCHO and H_2_O_2_ concentrations were examined to monitor HMHP formation in a wider concentration range. The concentrations were [HCHO]=[H_2_O_2_] = 1 mM, [HCHO]=[H_2_O_2_] = 10 mM, [HCHO]=[H_2_O_2_] = 30mM, and [HCHO]=[H_2_O_2_] = 80mM. Reactant concentrations were in the millimolar range due to the detection limit of the NMR system. Bis-HMHP, a biproduct of the reaction, was also formed at ∼6% concentration compared to HMHP. Formaldehyde was predominantly present as hydrate.

The concentration of HMHP was determined using the equilibrium constant of HMHP, which was obtained experimentally and calculated to be ${\text{K}}_{\text{eq}}=172(\pm 2){\;\text{M}}^{-1}$, comparable to the reported equilibrium constant by Zhao et al. (36) of${\;\text{K}}_{\text{eq}}=164{\;\text{M}}^{-1}$. The equilibrium constant is defined as

[4] $${\text{K}}_{\text{eq}}=\frac{{\left[\text{HMHP}\right]}_{\text{eq}}}{\left({\left[{\text{H}}_{2}{\text{O}}_{2}\right]}_{\text{o}}-{\left[\text{HMHP}\right]}_{\text{eq}}\right)\cdot \left({\left[\text{HCHO}\right]}_{\text{o}}-{\left[\text{HMHP}\right]}_{\text{eq}}\right)}$$

By solving Eq. **4**, the HMHP concentration was calculated.

In order to verify the calculated concentration, the ^1^H-NMR analysis was repeated using 3-(trimethylsilyl)-1-propanesulfonic acid sodium salt [${\left({\text{CH}}_{3}\right)}_{3}\text{Si}{\left({\text{CH}}_{2}\right)}_{3}{\text{SO}}_{3}\text{Na}$] as a standard compound. The equation used to calculate the HMHP concentration according to the area provided by the NMR spectrum and the standard compound used was

[5] $${\text{C}}_{\text{compound}}=\frac{{\text{I}}_{\text{compound}}}{{\text{N}}_{\text{compound}}}\cdot \frac{{\text{N}}_{\text{standard}}}{{\text{I}}_{\text{standard}}}\cdot {\text{C}}_{\text{standard}}\;,$$

where I is the integral area, N is the number of protons, and C the concentration of the compound of interest.

### Experimental Parameters of HMHP Equilibrium Kinetic Studies.

${\text{H}}_{2}{\text{O}}_{2}$ and HCHO solutions of the same concentration were mixed and analyzed using ^1^H-NMR for 37 time points in the range of 2 to 65 min to determine the rate constant, ${\text{k}}_{\text{f}}$, of HMHP formation. The reactions were rapid relative to the sample analysis time; therefore, a lower limit of the formation rate constant was estimated: $1.3\;\left(\pm 0.8\right){\;\text{M}}^{-1}\cdot {\text{s}}^{-1}$ (*SI Appendix*, Fig. S10). The upper bound of the formation rate constants, for our experimental conditions considering independent determination of the formation rate constant, was estimated equal to $100\;\left(\pm 35\right){\;\text{M}}^{-1}\cdot {\text{s}}^{-1}$.

The decomposition of HMHP was examined in a time range of 2 min to 10 d to determine the corresponding rate constant, ${\text{k}}_{\text{r}}$. A solution of HMHP was prepared and left for ∼12 h under dark conditions. HMHP was diluted in a ratio of 1:100 in order to initiate the decomposition. In addition, ${\text{k}}_{\text{r}}$ was also calculated using the equilibrium constant (Eq. **4**).

$${\text{H}}_{2}{\text{O}}_{2}(\text{aq})+\text{HCHO}(\text{aq})\overset{k_{f}}{\underset{k_{r}}{⇌}}\text{HMHP}$$

Different sets of HCHO and H_2_O_2_ concentrations, as described in *HMHP Synthesis and Measurement of HMHP Equilibrium Constant*, were examined.

### Model Parameters.

Version 12.2.1 of GEOS-Chem, a global chemical transport model that incorporates NASA Goddard Earth Observing System Fast Processing (GEOS-FP)–assimilated meteorological observations, was used to conduct the simulations (53–56). The model parameters for the chemistry of the oxidation of ${\text{HSO}}_{3}^{-}$ by ISOPOOH are described in detail in the work of Dovrou et al. (12). The original chemical mechanism was modified to include new isoprene chemistry (57) and the HMS chemistry described in the work of Moch et al. (35). Reactions involving HMHP were taken from Allen et al. (58), with oxidation rates and products from that study and formation yields from Nguyen et al. (59). Henry’s law constants for 1,2-ISOPOOH and 4,3-ISOPOOH, taken from Rivera-Rios (60), were computed as ${\text{K}}_{\text{H}}=\text{exp}(7761\cdot (1/\text{T})-14.415)\;\text{M}\cdot {\text{atm}}^{-1}$ and ${\text{K}}_{\text{H}}=\text{exp}(7843.4\cdot (1/\text{T})-16.953)\;\text{M}\cdot {\text{atm}}^{-1}$, respectively. The Henry’s law constant for lumped 1,4-ISOPOOH and 4,1-ISOPOOH was approximated as the average of the 1,2-ISOPOOH and 4,3-ISOPOOH values, although the small yields of the delta-ISOPOOH isomers make this value relatively unimportant. The Henry’s law constant for HMHP was approximated as $3.0\cdot 10^{6}\;\text{M}\cdot {\text{atm}}^{-1}$, an average of the experimental values from Staffelbach and Kok (61).

### Simulations.

Six simulations were performed to examine the effect of the oxidation reactions and the HCHO pathways described in this work. Simulation 1 incorporated the modifications described in “Model parameters” without making any additional changes to ${\text{SO}}_{2}$ chemistry and including the HMS chemistry (35). Simulation 2 included the oxidation of ${\text{HSO}}_{3}^{-}$ by HMHP and ISOPOOH (12) considering fast equilibrium: ${\text{k}}_{\text{f}}=100{\;\text{M}}^{-1}\cdot {\text{s}}^{-1}$ and ${\text{k}}_{\text{r}}=0.6{\;\text{s}}^{-1}$. Similar to simulation 2, simulations 3 and 4 included the same chemistry but with medium (${\text{k}}_{\text{f}}=1.3{\;\text{M}}^{-1}\cdot {\text{s}}^{-1}$ and ${\text{k}}_{\text{r}}=7.6\cdot 10^{-3}{\;\text{s}}^{-1}$) and slow (${\text{k}}_{\text{f}}=1.0\cdot 10^{-4}{\;\text{M}}^{-1}\cdot {\text{s}}^{-1}$ and ${\text{k}}_{\text{r}}=6\cdot 10^{-7}{\;\text{s}}^{-1}$) equilibria, respectively. Simulations 5 and 6 were sensitivity studies, where ${\text{k}}_{\text{f}}=100{\;\text{M}}^{-1}\cdot {\text{s}}^{-1}$, ${\text{k}}_{\text{r}}=7.6\cdot 10^{-3}{\;\text{s}}^{-1}$, and ${\text{k}}_{\text{r}}=6\cdot 10^{-7}{\;\text{s}}^{-1}$, respectively. The equations for the rate constants along with the mixing ratio of ${\text{H}}_{2}{\text{O}}_{2}$, ISOPOOH, and ${\text{O}}_{3}$ can be found in *SI Appendix*, Supplementary Text 4 and Figs. S6 and S7.

Simulations under “modern” conditions used biogenic emissions calculated from MEGAN 2.1 (62), biomass burning emissions from the Global Fire Emissions Database V4.1 inventory, anthropogenic emissions from the Community Emissions Data System global inventory with regional inventories based on the default GEOS-Chem setup, and fixed distributions of methane concentrations based on observations. Simulations under “preindustrial” conditions were performed by scaling global methane distributions down by 60% and removing all anthropogenic emissions. All simulations were run at 2° × 2.5° horizontal resolution with 72 vertical levels extending into the stratosphere and were initialized with a 1-y spin-up, after which annual and seasonal averages were derived from simulations with 2016 meteorology.

The regions examined were the Amazon (53.75 to 76.25°W, 11°S to 3°N), SE-US (81.25 to 93.75°W, 31 to 39°N), Congo Basin (11.25 to 28.75°E, 5°S to 5°N), East China (111.25 to 121.25°E, 23 to 41°N), India (68.75 to 88.75°E, 11 to 31°N), and Indonesia (96.25 to 148.75°E, 9°S to 5°N).

### Kinetic Chain Length.

The kinetic chain length of the catalytic role of HCHO was calculated as

[6] $$\text{Kinetic}\;\text{chain}\;\text{length}=\frac{{\text{k}}_{\left({\text{H}}_{2}{\text{O}}_{2}+\text{HCHO}\right)}\cdot \left[\text{HCHO}\right]\cdot [{\text{H}}_{2}{\text{O}}_{2}]}{{\text{k}}_{\left(\text{OH}+\text{HCHO}\right)}\cdot \left[\text{HCHO}\right]\cdot \left[\text{OH}\right]+{\text{k}}_{\left({\text{SO}}_{2}+\text{HCHO}\right)}\cdot \left[\text{HCHO}\right]\cdot [{\text{SO}}_{2,\text{aq}}]}.$$

From GEOS-Chem, the annual average values obtained after our simulations are ${\left[\text{HCHO}\right]}_{\text{g}}=3.99\;\text{ppbv}$, ${\left[{\text{H}}_{2}{\text{O}}_{2}\right]}_{\text{g}}=1.45\;\text{ppbv}$, and $[{\text{SO}}_{2,\text{aq}}]=0.045\;\text{ppbv}$.

The $\left[{\text{SO}}_{2,\text{aq}}\right]$ is two orders of magnitude lower compared to HCHO and H_2_O_2_; thus the kinetic rate of SO_2,aq_+HCHO is not considered.

For each equilibrium case, we calculated the kinetic chain length considering ${\text{k}}_{\text{f}}={\text{k}}_{\left({\text{H}}_{2}{\text{O}}_{2}+\text{HCHO}\right)}$ and ${\text{k}}_{\left(\text{OH}+\text{HCHO}\right)}=10^{9}{\;\text{M}}^{-1}{\text{s}}^{-1}$ (63). Arakaki et al. measured OH concentration in remote continental and remote marine regions equal to $8\cdot 10^{-15}\;\text{M}$ and $8\cdot 10^{-16}\;\text{M}$ (64).

For the medium equilibration case rate constant of this work ${\text{k}}_{\text{f}}=1.3{\;\text{M}}^{-1}{\text{s}}^{-1}$ and for the lower limit of the literature ${\text{k}}_{\text{f}}=7.5\cdot 10^{-2}{\;\text{M}}^{-1}{\text{s}}^{-1}$ and Henry’s law constant for H_2_O_2_ $10^{5}{\;\text{M}\;\text{atm}}^{-1}$, the kinetic chain length for remote continental regions was equal to maximum value 24 and minimum 1.5, respectively. The results show the catalytic nature of HCHO for the examined reactions. Considering fast equilibrium, the kinetic chain length is in the order of $10^{3}$, whereas for the slow equilibrium, the kinetic chain length is significantly low. Considering remote marine regions, the kinetic chain length increases by an order of magnitude.

When considering a closed system with liquid water content of $0.3{\;\text{g}\;\text{m}}^{-3}$ (cumulus cloud containing 800 droplets), the kinetic chain length values decrease by 40%. However, the decrease is not high, as the gas-phase mixing ratios are drawn down by the H_2_O_2_ partitioning to the condensed phase.

## Supplementary Material

Supplementary File

## Data Availability

The GEOS-Chem modifications have been implemented and are available at https://geos-chem.seas.harvard.edu/. All other study data are included in the article and/or *SI Appendix*.
