# Supplementary material for: Catalytic role of formaldehyde in particulate matter formation
Source: Proc Natl Acad Sci U S A. 2022 Jan 31;119(6):e2113265119. doi: 10.1073/pnas.2113265119 (PMC8833171; doi:10.1073/pnas.2113265119)
Supplement: Supplementary File [file pnas.2113265119.sapp.pdf]

SI Appendix

Catalytic Role of Formaldehyde in Particulate Matter Formation

Eleni Dovrou[a,&,*], Kelvin H. Bates[a], Jonathan M. Moch[b], Loretta J. Mickley[a], Daniel J. Jacob[a,b], Frank N. Keutsch[a,b,c,*]

[a]John A. Paulson School of Engineering and Applied Sciences, Harvard University, Cambridge, MA 02138, USA

[b]Department of Earth and Planetary Sciences, Harvard University, Cambridge, MA 02138, USA

[c]Department of Chemistry and Chemical Biology, Harvard University, Cambridge, MA 02138, USA

[&]Now at Multiphase Chemistry Department, Max Planck Institute for Chemistry, Mainz 55128, Germany

*Eleni Dovrou, Frank N. Keutsch.

Email: dovrouel@gmail.com (E.D.), keutsch@seas.harvard.edu (F.N.K.)

This PDF file includes:

Supplementary Text
Supplementary Discussion
Figures S1 to S12
Tables S1 to S10

Supplementary Text

1: Definition of $SO_{2,aq}$ and HCHO used in this work

The term $SO_{2,aq}$ used in this work refers to the S(IV) that is present in cloud and fog water. The typical cloud and fog water pH range is 3-6, in which $SO_{2,aq}$ is in the form of bisulfite (HSO_3^-). When pH≳6 $SO_{2,aq}$ is in the form of sulfite (SO_3^{2-}).

The term HCHO refers to the free HCHO plus its hydrated form, which is the dominant form in a cloud and fog droplet.

2: Second order reaction solution model

The rate of oxidation of $SO_{2,aq}$ by the examined peroxides is:

$$\frac{d[\mathrm{peroxide}]}{dt} = k \cdot [HSO_3^-] \cdot [\mathrm{peroxide}] \qquad \text{(S1)}$$

where k is the second order rate constant of the reaction and $[HSO_3^-]$, $[\mathrm{peroxide}]$ are the concentrations of $SO_{2,aq}$ and the examined peroxides. The pH remained constant for each pH value examined, thus $[H^+]$ is considered constant.

By solving the differential equation, we have:

$$[\mathrm{peroxide}]_i = \frac{[HSO_3^-]_o - [\mathrm{peroxide}]_o}{\left(\frac{[HSO_3^-]_o}{[\mathrm{peroxide}]_o}\right) * e^{k \cdot t_i \cdot ([HSO_3^-]_o - [\mathrm{peroxide}]_o)} - 1} \qquad \text{(S2)}$$

By conservation of mass from the initial peroxide concentration, the concentration of sulfate is:

$$[SO_4^{2-}]_i = 15 \cdot 10^{-6} - [\mathrm{peroxide}]_i \qquad \text{(S3)}$$

3: Calculation of uncertainty

In order to calculate the uncertainty of the rate constants, weighted nonlinear regression was applied to the data followed by a Monte Carlo analysis. The analysis was conducted by repeating the fitting procedure of the model 5000 times. A set of values were randomly sampled considering normal distribution and 95% confidence interval was selected. The standard deviation represents the uncertainty due to the model fit.

The standard deviation, S_i, is defined as $S_i = \frac{1}{N-1} \cdot \sum_{i=1}^{N} |C_i - \mu|^2$, where N is the number of the experimental concentrations, C_i is the concentration i and μ is the mean value of the concentrations and is equal to $\mu = \frac{1}{N} \cdot \sum_{i=1}^{N} C_i$.

The uncertainty in this work represents the precision of the measurements and not the accuracy, due to unknown systematic errors.

4: Rate constant equations used in GEOS-Chem

pH > 4.5:

$$k_{H_2O_2} = (5.3 \cdot 10^{19-2 \cdot pH}) \cdot (\exp(-4.76 \cdot 10^3/T)/1.1653 \cdot 10^{-7}) \qquad \text{(S4)}$$

$$k_{1,2-ISOPOOH} = (1.0 \cdot 10^3 + 0.65 \cdot 10^3 \cdot (pH - 4.5)) \cdot (\exp(-4.76 \cdot 10^3/T)/1.1653 \cdot 10^{-7}) \tag{S5}$$

$$k_{4,3-ISOPOOH} = (0.9 \cdot 10^2 + 0.9 \cdot 10^2 \cdot (pH - 4.5)) \cdot (\exp(-4.76 \cdot 10^3/T)/1.1653 \cdot 10^{-7}) \tag{S6}$$

$$k_{HMHP} = (1.5 \cdot 10^4 + 7.3 \cdot 10^3 \cdot (pH - 4.5)) \cdot (\exp(-4.76 \cdot 10^3/T)/1.1653 \cdot 10^{-7}) \tag{S7}$$

pH < 4.5:

$$k_{H_2O_2} = (5.3 \cdot 10^{13-\frac{pH}{1.5}}) \cdot (\cdot \exp(-4.76 \cdot 10^3/T)/1.1653 \cdot 10^{-7}) \tag{S8}$$

$$k_{1,2-ISOPOOH} = (1.0 \cdot 10^3) \cdot (\exp(-4.76 \cdot 10^3/T)/1.1653 \cdot 10^{-7}) \tag{S9}$$

$$k_{4,3-ISOPOOH} = (0.9 \cdot 10^2 + 1.33 \cdot 10^2 \cdot (4.5 - pH)) \cdot (\exp(-4.76 \cdot 10^3/T)/1.1653 \cdot 10^{-7}) \tag{S10}$$

$$k_{HMHP} = (1.5 \cdot 10^4 + 6.7 \cdot 10^2 \cdot (pH - 4.5)) \cdot (\exp(-4.76 \cdot 10^3/T)/1.1653 \cdot 10^{-7}) \tag{S11}$$

Supplementary Discussion

1: Comparison of HMS with other aldehyde-S(IV) species

In cloud and fog water HCHO can participate in two pathways contributing to PM formation: production of SO_4^{2-} and of sulfur-based organic compounds, such as HMS. Previous studies examined the formation rates of aldehyde-S(IV) species, which have faster formation rates at pH≥4.5 than HMS. Although the solubility of these aldehydes is comparable to that of HCHO, their concentrations in the atmosphere are lower[1-3]. The ambient concentration range of HCHO in the atmosphere is 0.5-15 ppbv, with the higher values corresponding to urban and polluted regions, whereas the ambient concentrations of the aldehydes examined in previous studies are on the order of 10-100 pptv in remote regions and up to 10 ppbv in polluted regions[4-6]. HMS formation depends on the presence of HCHO and even though some aldehyde-S(IV) can have higher formation rates, up to a factor of 4, the concentration of HCHO is generally higher which constitutes HMS as the most important aldehyde-S(IV) compound. In addition, HMS is an important single carbon S(IV) compound which can contribute to global and regional sulfur budget in PM[7-8].

2: Comparison of methods used to determine the equilibrium, formation and decomposition constants of HMHP

The equilibrium constant of HMHP presented in this work and in the literature are in agreement, within uncertainty[9-11]. However, the formation and decomposition rate constants reported in the literature differ by orders of magnitude[9-11]. To be more specific, Marklund (1971) and Zhou and Lee (1992) reported values that are different by approximately 2 orders of magnitude, while the values reported herein are significantly higher and lower, for the case of fast and slow equilibrium, respectively, and comparable with the highest value reported in the literature, for the case of medium equilibrium (Table S1). Marklund (1971) synthesized HMHP in a petri dish containing HCHO and H_2O_2 at a ratio of 1:1. The quantification was achieved via absorption spectroscopy, calculating the total peroxide concentration. Calibration was achieved via absorption of H_2O_2 after addition of Ti(IV)[10]. Zhou and Lee (1992) used an amperometric technique in which electrodes were conditioned in HCHO solution and H_2O_2 was added to form HMHP. HCHO was in large excess in order to follow a pseudo-first order reaction and the H_2O_2 concentration was measured to

determine the rate constants. HMHP and bis-HMHP were calibrated as H_2O_2. Therefore, in both studies H_2O_2 was the compound traced to determine the formation and decomposition of HMHP.

In this work, the method described by Zhao et al. (2013) to synthesize HMHP in water was followed [9]. Zhao et al. (2013) report the equilibrium constant without quantifying the formation and decomposition rate constants. In the work presented herein, the HMHP concentration was used to determine the formation and decomposition rate constants.

3: α- and β-hydroxyhydroperoxides

Comparing SO_2 oxidation by HMHP and ISOPOOH, HMHP oxidizes SO_2 significantly faster than ISOPOOH (Table S3). HMHP is an α-hydroxyhydroperoxide (its the hydroxyl and hydroperoxide groups are on the same carbon) whereas ISOPOOH is a β-hydroxyhydroperoxide (the two functional groups are present on neighboring carbons; Fig. S11). Therefore, given the higher oxidation rate constant observed for HMHP than for ISOPOOH in our work, it may be generally true that α-hydroxyhydroperoxides are more reactive than β-hydroxyhydroperoxides.

4: The role of cloud pH

Cloud pH determines the ability of $SO_{2,aq}$ to react with HCHO or to be oxidized forming sulfate. H_2O_2 and HMHP can oxidize $SO_{2,aq}$ rapidly within the cloud pH range of 3-6 and the rate of oxidation increases significantly under acidic conditions. At pH=4.5 and 3 the rate constant of the reaction of H_2O_2+$SO_{2,aq}$ increases by 2 and 3 orders of magnitude, respectively, compared to pH=5.5 [12]. Similar trend is observed in the oxidation of $SO_{2,aq}$ by HMHP; however, the reaction is faster compared to H_2O_2+$SO_{2,aq}$ and a lower limit of the rate constants were obtained. Thus, the magnitude of increase of the rate constant under acidic (pH<4.5) conditions is estimated to be at least an order of magnitude higher compared to pH≥4.5. In contrast, ISOPOOH oxidizes $SO_{2,aq}$ at the same rate, within uncertainty, with H_2O_2 at pH=5.5 but, at lower pH, ISOPOOH has a lower oxidation rate[13]. Finally, another important oxidant of $SO_{2,aq}$ at cloud pH>4 is O_3, with rate constant 2 and 1 orders of magnitude higher compared to H_2O_2 and ISOPOOH and HMHP, respectively[13-14].

Oxidation of $SO_{2,aq}$ results in sulfate formation, contributing to the sulfur budget of PM. However, formation of HMS is also important for the sulfur PM budget. In-cloud HMS formation is 10 times faster at pH=5 compared to pH=4 and its decomposition is an order of magnitude faster at pH=5.5 Rao and Collett (1995) also showed that at pH<5 HMS formation is slow but at pH>5 it can be competitive with the oxidation of H_2O_2 and O_3[14]. Highlighting the importance of HMS, Rao and Collett (1995) provided evidence that HMS formation and the reaction of O_3+ $SO_{2,aq}$ become more important than the reaction of H_2O_2+ $SO_{2,aq}$ when pH>6.4 However, at pH>6 HMS becomes unstable and can be oxidized by OH and H_2O_2[15-18]. Rao and Collett (1995) observed that HCHO concentration can also affect the rate of the reactions[14]. At high aqueous HCHO concentration, HMS formation and $SO_{2,aq}$ oxidation by O_3 can occur at similar rates, whereas at low HCHO and H_2O_2 concentrations, HCHO is more likely to be oxidized by OH than with $SO_{2,aq}$, forming HMS.

5: Reaction of catalase with HMHP

Catalase is an enzyme that is widely used, due to its high specificity, to consume H_2O_2[12, 19-20]. However, catalase has been shown to react in a slower rate with simple organic peroxide consumption, i.e. methyl hydroperoxide, peracetic acid and ethyl hydroperoxide[20]. The effect of catalase in multifunctional organic peroxides is uncertain. Dovrou et al. (2019) use this enzyme to quench ISOPOOH, as experimental analysis revealed its rapid reaction with ISOPOOH[13]. In this work, the reaction of catalase with HMHP was evaluated, showing rapid consumption of HMHP within the analysis time.

6: HMHP vs terpene a-HHP decomposition in water

The equilibration rate of HMHP is inversely dependent on [H^+], as it increases with increasing pH (base catalysis)[9,11]. Therefore, the rate of formation and decomposition of HMHP decreases under acidic conditions. Qiu et al. (2020) observed an opposite trend for terpene derived a-hydroxyalkyl-hydroperoxides (a-HHP)[21]. a-HHP decomposition accelerates under acidic conditions, revealing its potential importance in regions where cloud water pH is lower than 4, i.e. Asia[22]. The difference in the pH dependence of a-hydroperoxide decomposition could result from differences in their properties and structure and resulting reactivity. Both hydroperoxides are highly water soluble, however, HMHP is a one carbon molecule in contrast to a-HHP which is more complex with two hydroxyl and one hydroperoxide groups. The high reactivity of HMHP and a-HHP may result from the structural connectivity of their reactive groups: one hydroxyl and one hydroperoxide group are attached to the same carbon. The additional hydroxyl group and the carbonyl group in the a-HHP structure could favor faster decomposition under acidic conditions. However, further investigation is required, which is out of the scope of the present study.

Supplementary reference

1. Olson, T. M., Torry, L. A., Hoffmann, M. R. Kinetics of the formation of hydroxyacetaldehyde-sulfur(IV) adducts at low pH. *Environ. Sci. Technol.* 22, 1284–1289 (1988).

2. Olson, T. M., Hoffmann, M. R., Kinetics, mechanism, and thermodynamics of glyoxal-S(IV) adduct formation. *J. Phys. Chem.* 92, 533–540 (1988).

3. Olson, T. M., Hoffmann, M. R., Hydroxyalkylsulfonate formation: Its role as a S(IV) reservoir in atmospheric water droplets. 23, 985–997 (1989).

4. Salthammer, T. Formaldehyde in the ambient atmosphere: From an indoor pollutant to an outdoor pollutant? *Angew. Chemie - Int. Ed.* 52, 3320–3327 (2013).

5. Stönner, C., Derstroff, B., Klüpfel, T., Crowley, J. N., Williams, J., Glyoxal measurement with a proton transfer reaction time of flight mass spectrometer (PTR-TOF-MS): characterization and calibration. *J. Mass Spectrom.* 52, 30–35 (2017).

6. Li, M., Li, Q., Nantz, M. H., Fu, X. A., Analysis of Carbonyl Compounds in Ambient Air by a Microreactor Approach. *ACS Omega* 3, 6764–6769 (2018).

7. Moch, J. M., Dovrou, E., Mickley, L. J., Keutsch, F., Cheng, Y., Jacob, D. J., Jiang, J., Li, M., Munger, J. W., Qiao, X., Zhang, Q., Contribution of Hydroxymethane Sulfonate to Ambient Particulate Matter: A Potential Explanation for High Particulate Sulfur During Severe Winter Haze in Beijing. *Geoph. Res. Lett.* 45, 11969–11979 (2018).

8. Moch, J. M., Dovrou, E., Mickley, L. J., Keutsch, F. N., Liu, Z., Wang, Y., Dombek, T. L., Kuwata, M., Budisulistriorini, S. H., Yang, L., Decesari, S., Paglione, M., Alexander, B., Shao, J., Munger, J. W., Jacob, D. J., Global importance of hydroxymethanesulfonate in ambient particular matter: Implications for air quality. *J. Geoph. Res. Atmospheres.* 125, 1-14 (2020).

9. Zhao, R., Lee, A. K. Y., Soong, R., Simpson, A. J., Abbatt, J. P. D., Formation of aqueous-phase α-hydroxyhydroperoxides (α-HHP): Potential atmospheric impacts. *Atmos. Chem. Phys.* 13, 5857–5872 (2013).

10. Marklund, S. The simultaneous determination of bis(hydroxymethyl)-peroxide (BHMP), hydroxymethylhydroperoxide (HMP), and H_2O_2 with titanium(IV). Equilibria between the peroxides and the stabilities of HMP and BHMP at physiological conditions. *Acta Chemica Scandinavica* 25, 3517–3531 (1971).

11. Zhou, X., Lee, Y. N., Aqueous solubility and reaction kinetics of hydroxymethyl hydroperoxide. *J. Phys. Chem.* 96, 265–272 (1992).

12. Lind, J. A., Lazrus, A. L., Kok, G. L., Aqueous Phase Oxidation of Sulfur(IV) by Hydrogen-Peroxide, Methylhydroperoxide, and Peroxyacetic Acid. *J. Geophys. Res.* 92, 4171–4177 (1987).

13. Dovrou, E., Rivera-Rios, J. C., Bates, K. H., Keutsch, F. N., Sulfate Formation via Cloud Processing from Isoprene Hydroxyl Hydroperoxides (ISOPOOH). *Environ. Sci. Technol.* 53, 12476-12484 (2019)

14. Rao, X. & Collett, J. L. Behavior of S(IV) and Formaldehyde in a Chemically Heterogeneous Cloud. *Environ. Sci. Technol.* 29, 1023–1031 (1995).

15. Kok, G. L., Gitlin, S. N., Lazrus, A. L., Kinetics of the Formation and Decomposition of Hydroxymethanesulfonate. *J. Geoph. Res.* 91, 2801–2804 (1986).

16. Martin, L. R., Easton, M. P., Foster, J. W., Hill, M. W., Oxidation of hydroxymethanesulfonic acid by Fenton's reagent. *Atmos. Environ.* 23, 563–568 (1989).

17. Chapman, E. G., Barinaga, C. J., Udseth, H. R., Smith, R. D., Confirmation and quantitation of hydroxymethanesulfonate in precipitation by electrospray ionization-tandem mass spectrometry. *Atmos. Environ.* 24A, 2951–2957 (1990).

18. Dovrou, E., Lim, C. Y., Canagaratna, M. R., Kroll, J. H., Worsnop, D. R., Keutsch, F. N. Measurement techniques for identifying and quantifying hydroxymethanesulfonate (HMS) in an aqueous matrix and particulate matter using aerosol mass spectrometry and ion chromatography. *Atmos. Meas. Tech.* 12, 5303–5315 (2019).

19. A. Mhamdi, *et al.*, Catalase function in plants: A focus on Arabidopsis mutants as stress-mimic models. *J. Exp. Bot.* 61, 4197–4220 (2010).

20. M. M. Palcic, H. B. Dunford, The reaction of human erythrocyte catalase with hydroperoxides to form compound I. *J. Biol. Chem.* 255, 6128–6132 (1980).

21. J. Qiu, K. Tonokura, S. Enami, Proton-Catalyzed Decomposition of α-Hydroxyalkyl-Hydroperoxides in Water. *Environ. Sci. Technol.* 54, 10561–10569 (2020).

22. Shah, V., Jacob, D. J., Moch, J. M., Wang, X., Zhai, S., Global modeling of cloud water acidity, percipitation acidicty, and acid inputs to ecosystems. *Atmos. Chem. Phys.* 20, 12223-12245 (2020).

Figures

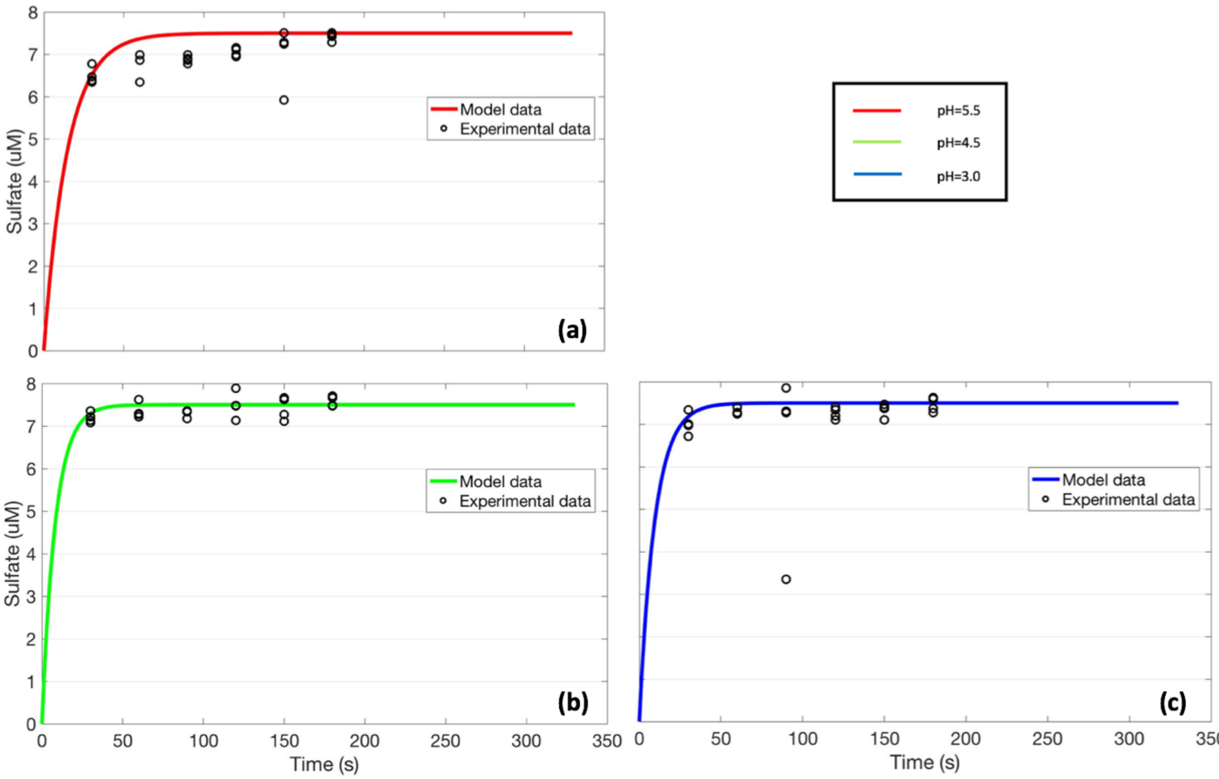

Figure S1. Sulfate production. Production of sulfate vs time due to the oxidation of $SO_{2,aq}$ via HMHP for (a) pH=5.5, (b) pH=4.5 and (c) pH=3. The red, green and blue lines represent the second order solution model for pH=5.5, 4.5 and 3 respectively. The uncertainty in concentration of a single measurement is 0.1 μM (Supplementary Text 3).

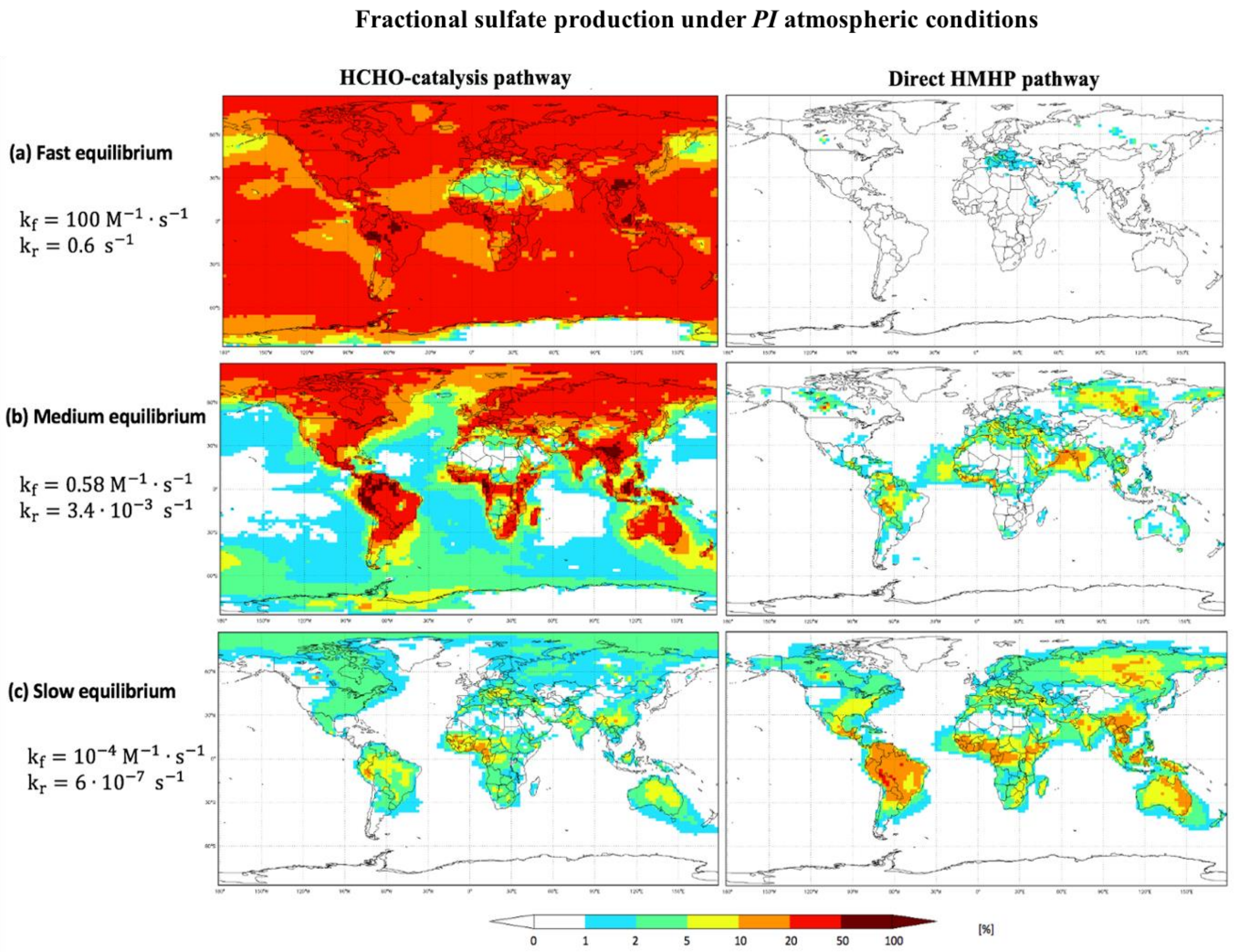

Figure S2. Contribution of the two HMHP pathways to column sulfate production. The contribution is estimated for fast, medium and slow HMHP equilibrium under pre-industrial atmospheric conditions, according to the GEOS-Chem simulations at altitude 0-10 km. If the equilibrium is reached under the fast rate, the HCHO-catalysis pathways is dominant compared to the direct HMHP pathway for the oxidation of dissolved SO_2.

A **Percent change of sulfate production and SO_2 lifetime under *PI* atmospheric conditions**

B **Percent change of HMS production under *PI* atmospheric conditions**

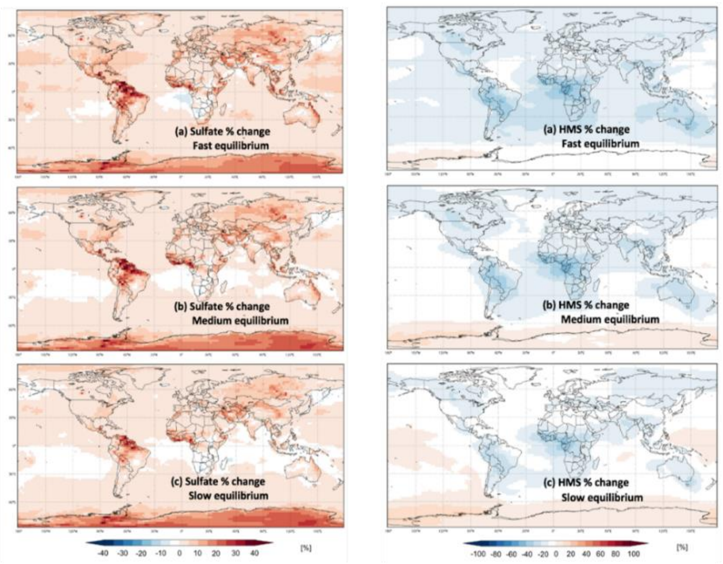

C

Ratio of SO_2 lifetime, τ_{SO_2}, of the value obtained from the equilibrium cases to the base simulation.

	Fast HMHP equilibrium $\frac{(\tau_{SO_2})_{equil.}}{(\tau_{SO_2})_{base}}$	Medium HMHP equilibrium $\frac{(\tau_{SO_2})_{equil.}}{(\tau_{SO_2})_{base}}$	Slow HMHP equilibrium $\frac{(\tau_{SO_2})_{equil.}}{(\tau_{SO_2})_{base}}$
**Global**	93%	98%	100%
**SE-US**	76%	82%	91%
**Amazon**	55%	54%	65%
**Congo Basin**	70%	73%	81%
**Indonesia**	89%	92%	94%

D

Ratio of the parameter p, defined as $p = \frac{HMS\ production}{Sulfate\ production}$, of the value obtained from the equilibrium cases to the base simulation.

	Fast HMHP equilibrium $\frac{p_{equil.}}{p_{base}}$	Medium HMHP equilibrium $\frac{p_{equil.}}{p_{base}}$	Slow HMHP equilibrium $\frac{p_{equil.}}{p_{base}}$
**Global**	56%	59%	62%
**SE-US**	12%	13%	15%
**Amazon**	17%	17%	22%
**Congo Basin**	15%	15%	20%
**Indonesia**	13%	14%	15%

Figure S3. Percent change in column sulfate and HMS production and ratio of SO_2 lifetime and HMS to sulfate production. Like Fig. 3 in the main manuscript, Fig. S3 presents the percent change in column sulfate (A) and HMS (B) production considering fast, medium and slow HMHP equilibrium under pre-industrial atmospheric conditions at altitude of 0-10 km. The GEOS-Chem model described in this work was used to perform the simulations. The percentages presented in the table (C) represent the ratio of SO_2 lifetime, τ_{SO_2}, of the value obtained from the equilibrium cases to that from the base simulation for the Amazon, Southeastern United States, Congo Basin and Indonesia. The percentages are all lower than 100%; thus, the equilibrium cases reduce the SO_2 lifetime. The percentages presented in the table (D) represent the ratio of the parameter $p = \frac{\text{production (HMS)}}{\text{production }(SO_4^{2-})}$ of the value obtained from the equilibrium cases to that from the base simulation for the Amazon, Southeastern United States, Congo Basin and Indonesia. The percentages are all lower than 100%; thus, the equilibrium cases reduce the production of HMS compared to sulfate. (Tables S5-S7)

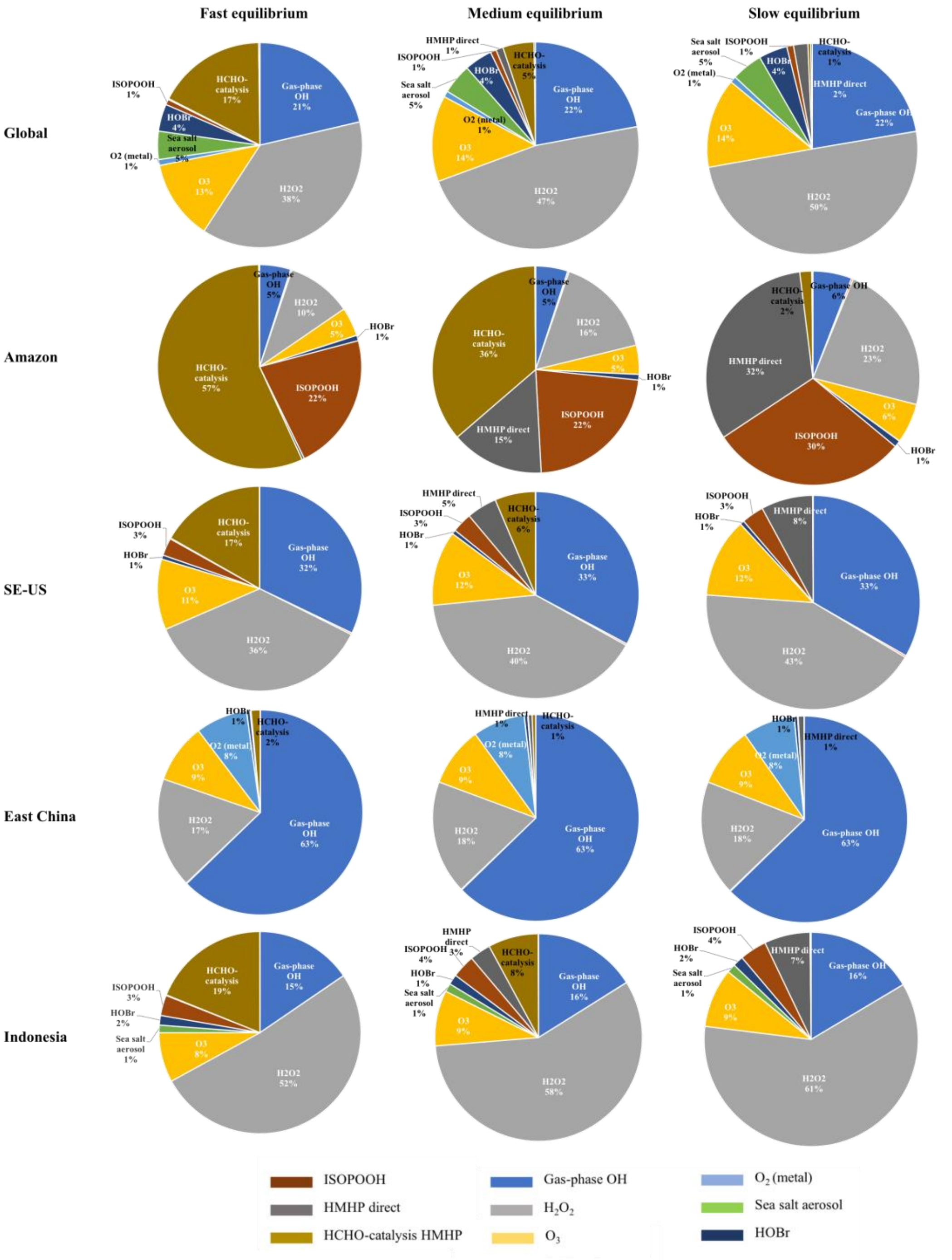

Figure S4. Contribution to sulfate production of the oxidative pathways included in GEOS-Chem simulations at global and regional scales at altitude of 0-10 km. The values reported represent annual averages under current atmospheric conditions at each of the three HMHP equilibrium cases.

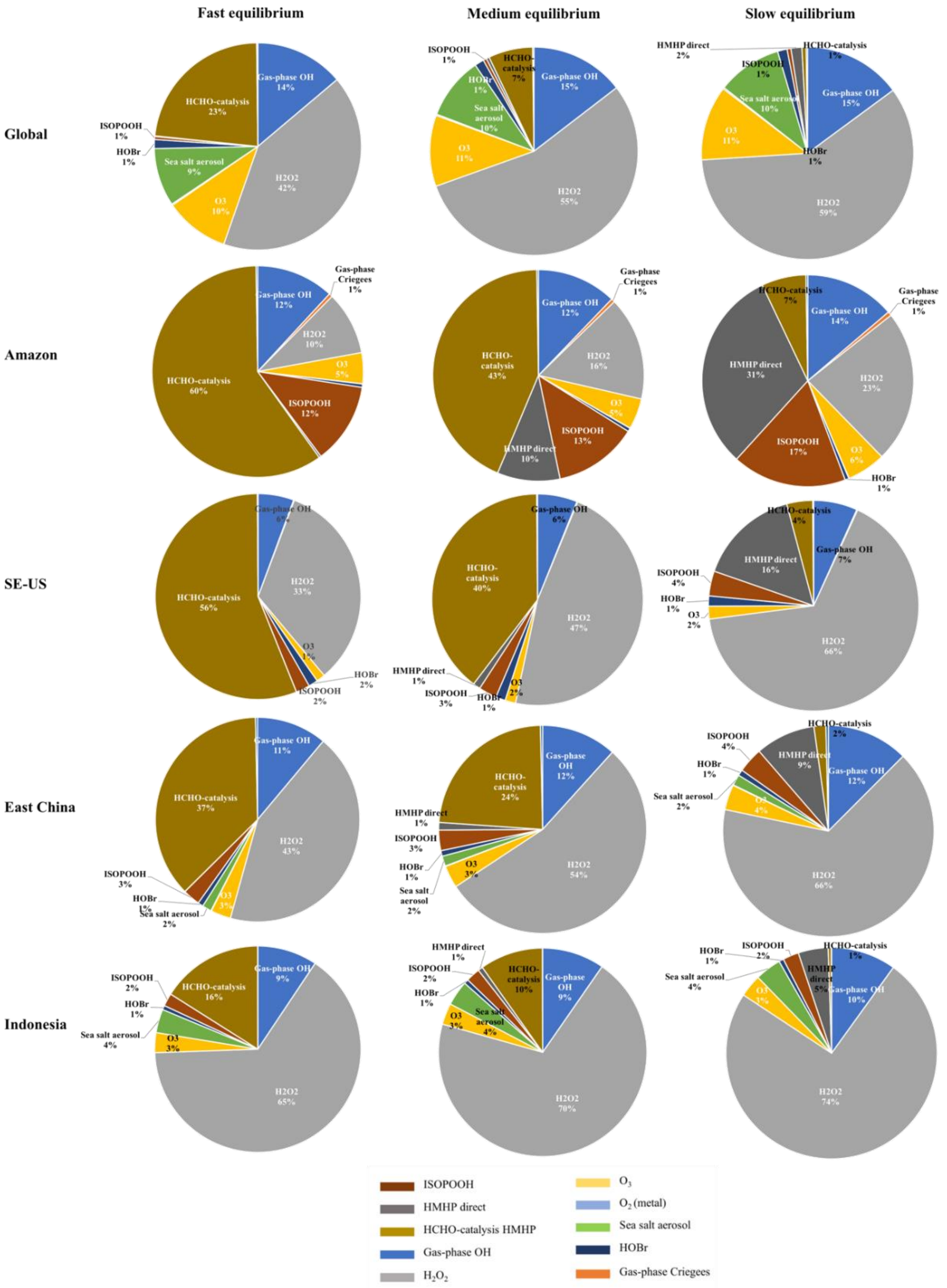

Figure S5. Contribution of sulfate production by the oxidative pathways included in GEOS-Chem simulations under pre-industrial atmospheric conditions at altitude of 0-10 km. The values reported represent annual averages under pre-industrial atmospheric conditions at each of the three HMHP equilibrium cases.

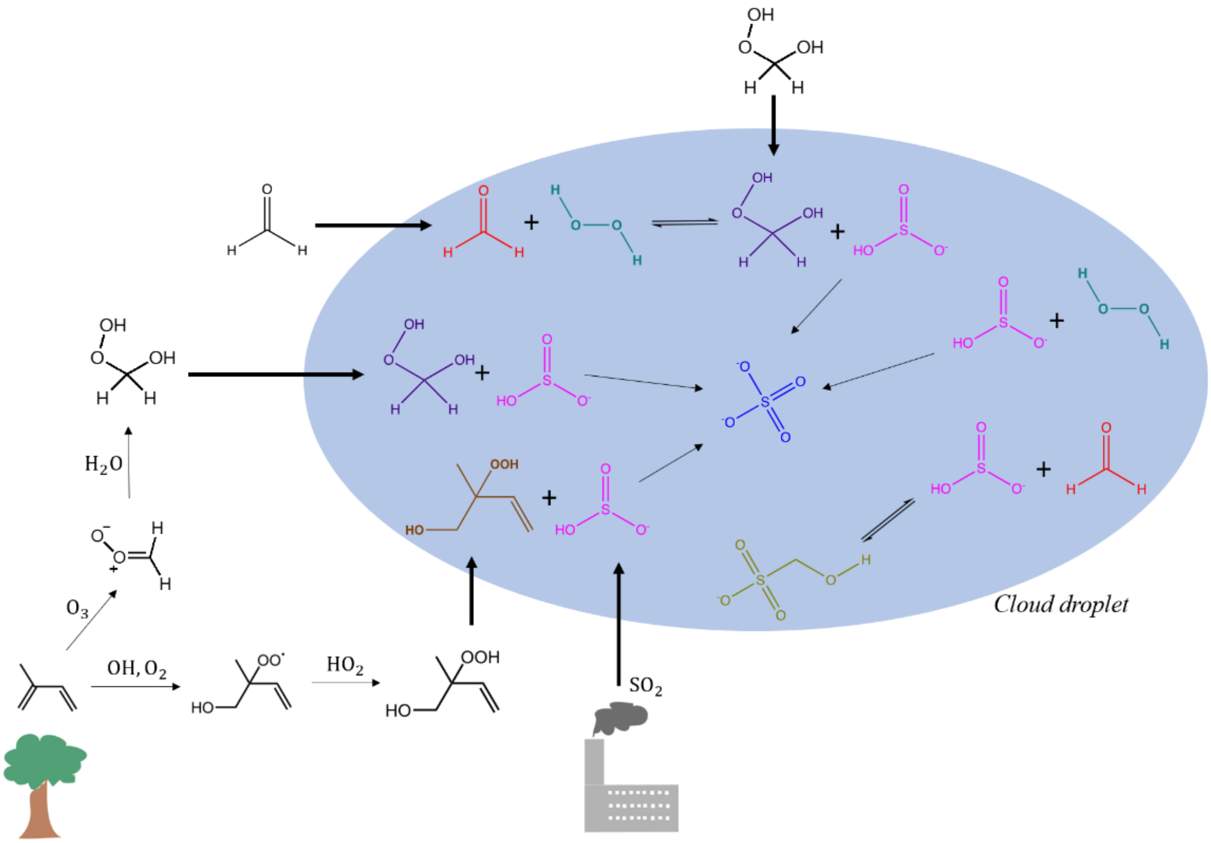

Figure S6. Schematic of the oxidation pathways of dissolved SO_2 by multifunctional organic hydroperoxides. The HCHO pathways are also presented showing the importance of HCHO in aqueous-phase sulfur chemistry. The catalytic reformation of HCHO is shown in Figure 4(A).

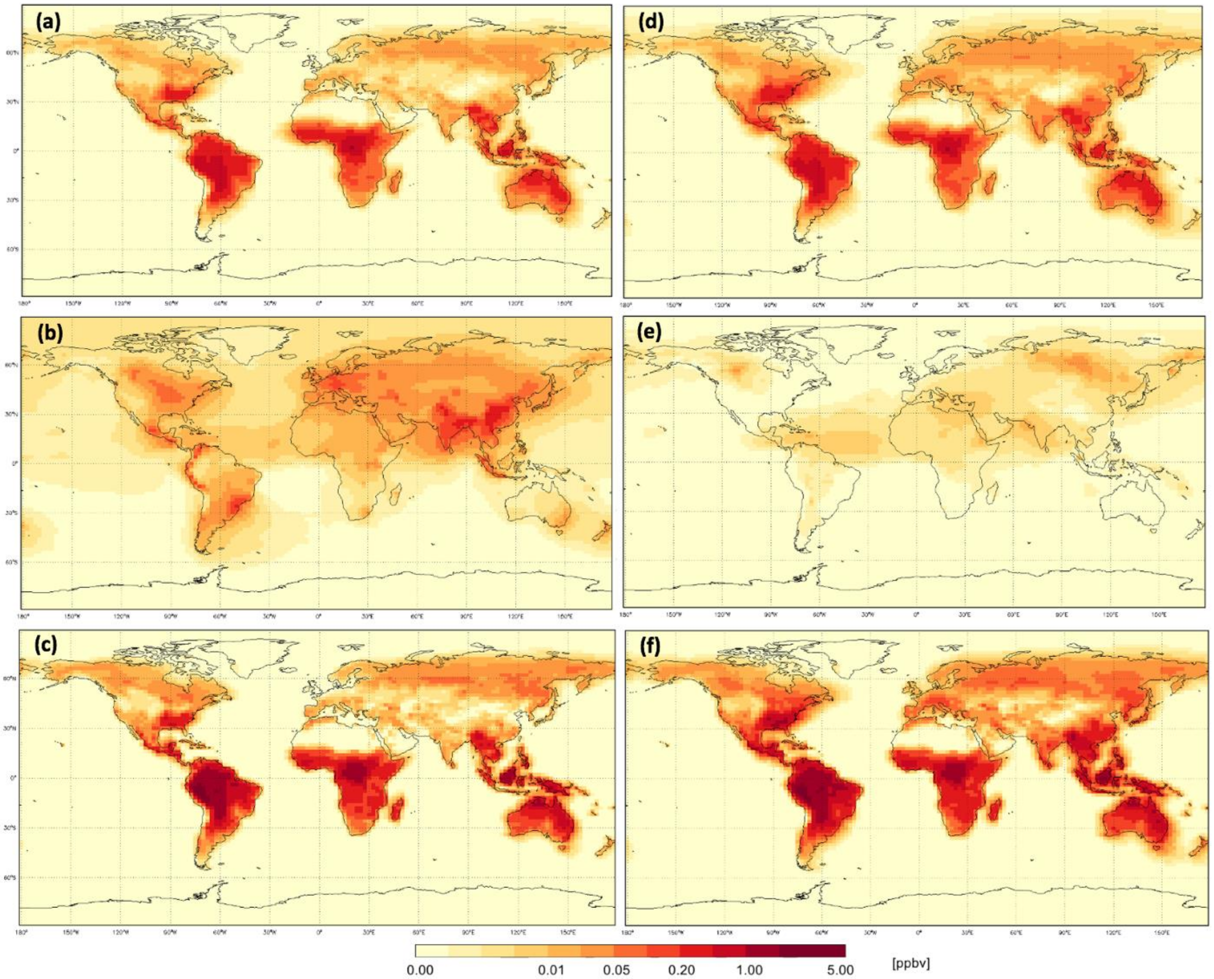

Figure S7. Annual boundary mixing ratios. Average annual boundary layer mixing ratios of HMHP (a,d), HMS (b,e) and ISOPOOH (c,f) under current (a,b,c) and pre-industrial atmospheric conditions (d,e,f) calculated according to the GEOS-Chem simulations at altitude 0-10 km.

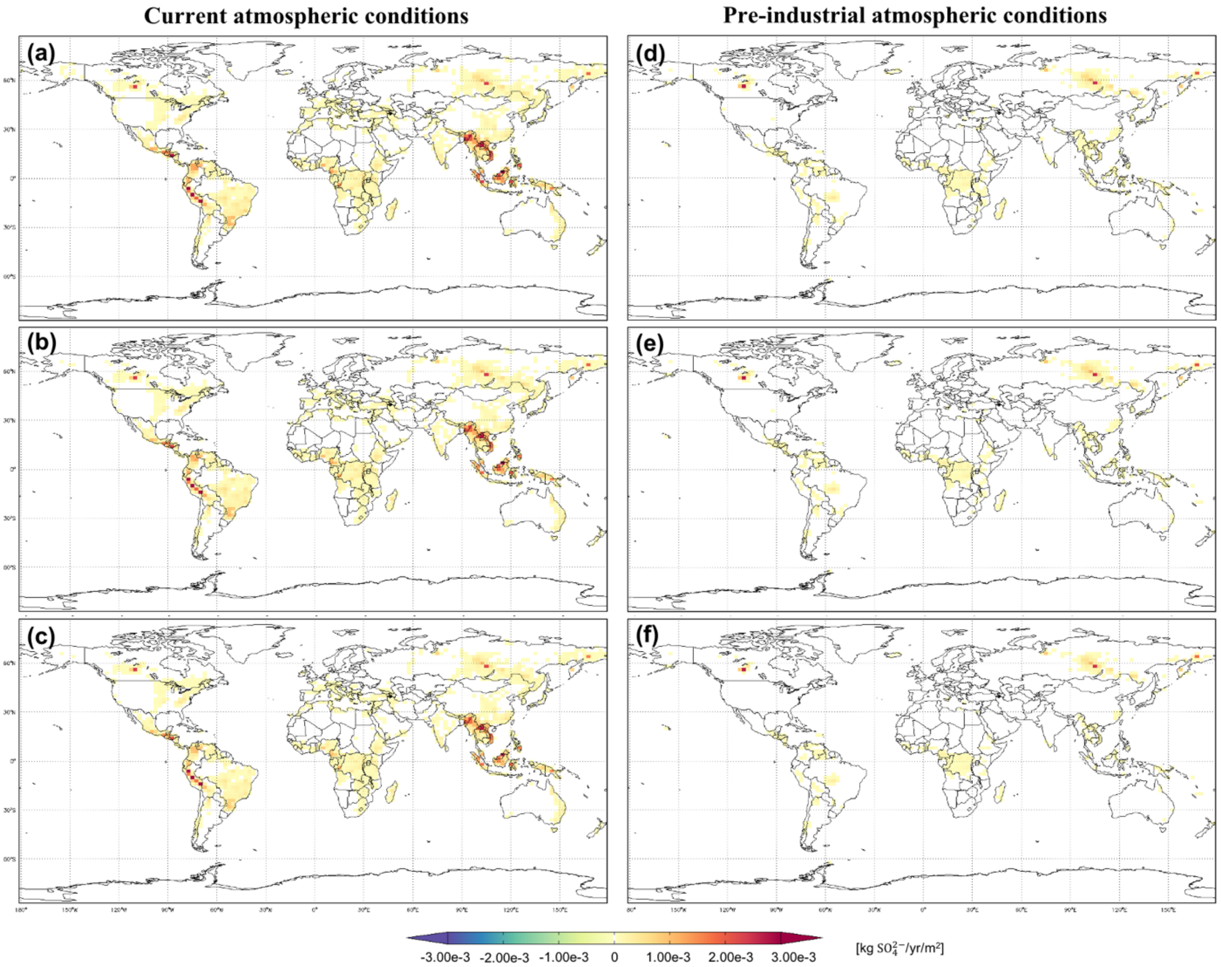

Figure S8. Absolute difference between base and HMHP equilibrium case in annual column sulfate production at altitude 0-10 km. The difference is presented for (a,d) fast HMHP equilibrium, (b,e) median HMHP equilibrium and (d,f) slow HMHP equilibrium under both current and pre-industrial atmospheric conditions.

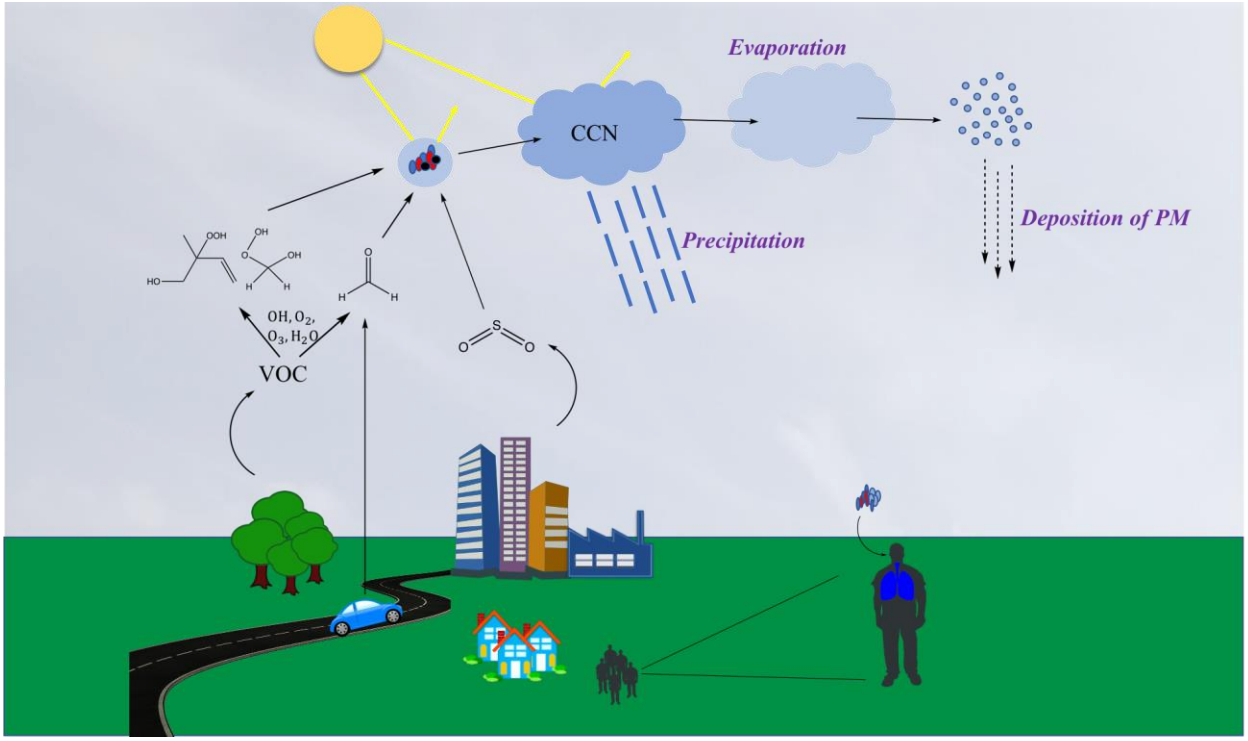

Figure S9. Schematic representation of the chemical procedures described in this work and their effects on Earth's climate and human health. Once clouds are formed, they will either result in (1) precipitation or (2) evaporation, leaving PM in the atmosphere. PM can be transported or move down to the surface, entering the human respiratory tract and infiltrating the ground and water sources.

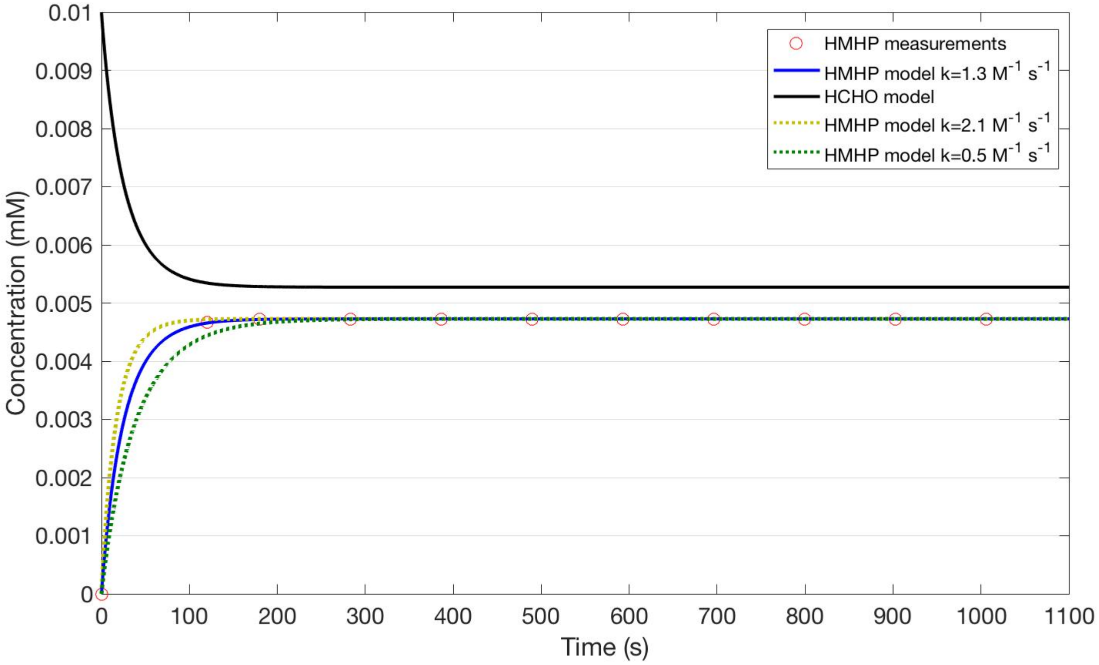

Figure S10. Formation of HMHP and decomposition of HCHO. The formations were simulated using the numerical solution of the rate of HMHP formation (Equation 3) in order to represent the HMHP measurements obtained via the ^{1}H-NMR analysis. (see Materials and Methods) The HMHP formation rate constant used in the simulation was $1.3\ (\pm 0.8)\ \mathrm{M}^{-1}\cdot\mathrm{s}^{-1}$ and the initial concentrations of the reactants were [HCHO]=[H_2O_2]=10 mM. The solution was at 25°C and pH=5.5. The measured HMHP concentrations were replicated four times over the time range of 2-65 min. The values were on the plateau of the reaction; thus, the timescale of 0-25 min is presented.

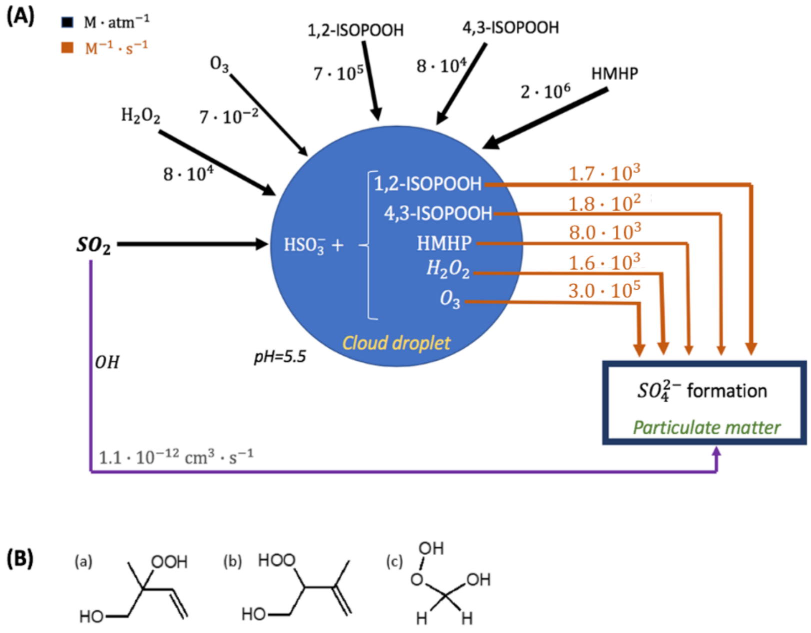

Figure S11. Examined oxidative reactions and hydroperoxides. (A) Summary of the oxidative reactions of sulfur dioxide in the gas[13] and aqueous phases (values are for pH=5.5). (B) Chemical structures of the two main ISOPOOH isomers, 1,2-ISOPOOH (a) and 4,3-ISOPOOH (b) and HMHP (c).

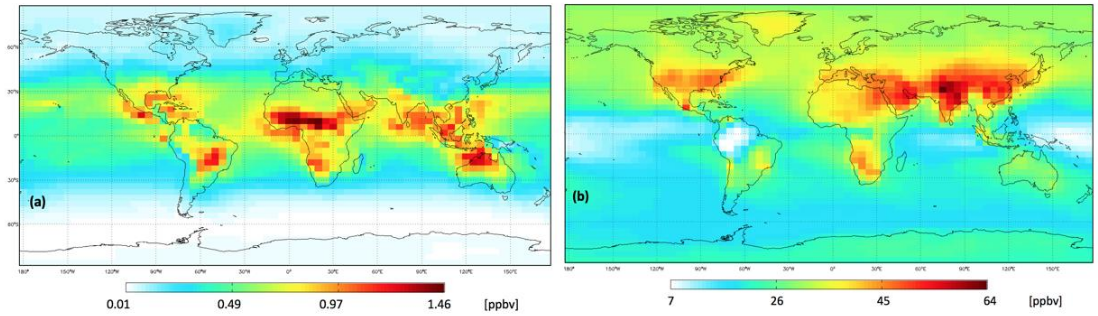

Figure S12. Boundary mixing ratios of H_2O_2 and O_3. Average annual boundary layer mixing ratios of (a) H_2O_2 and (b) O_3 calculated according to the GEOS-Chem simulations at altitude 0-10 km.

Tables

Table S1. Equilibrium constant, and formation and decomposition rate constants of the HCHO + H2O2 ⇌ HMHP reaction, reported in this work and the literature[9-11].

	$\mathbf{K_{eq}}\ (\mathbf{M^{-1}})$	$\boldsymbol{k_f}\ (\mathbf{M^{-1}\cdot s^{-1}})$	$\boldsymbol{k_r}\ (\mathbf{s^{-1}})$
**Marklund, 1971**	126±6	$7.5 \cdot 10^{-2}$	$5.9 \cdot 10^{-4}$
**Zhou and Lee, 1992**	149	$9.4 \cdot 10^{-1}$	$6.3 \cdot 10^{-3}$
**Zhao et al., 2013**	164±31	-	-
**This work**	172±2	$k_{f,meas} = 1.3$<br>$k_{f,calc} = 1.0 \cdot 10^{-4}$	$k_{r,meas} = 6.0 \cdot 10^{-7}$<br>$k_{r,calc} = 7.6 \cdot 10^{-3}$

Table S2. Experimental parameters of the oxidation of $SO_{2,aq}$ by HMHP.

**Parameter**	**Value**	**Units**
**[$SO_{2,aq}$]**	15	μM
**[peroxide]**	7.5	μM
**Examined pH values**	3, 4.5 and 5.5	-
**Examined temperature**	25	ºC

Table S3. Second order rate constants of the reaction of $SO_{2,aq}$+H_2O_2 reported in the literature.

**Source**	**Rate constant ($\mathbf{M^{-1}\cdot s^{-1}}$) @25 ºC**
**Hoffman and Edwards, 1975**	$2.0 \cdot 10^{2}$
**Hegg and Hobbs, 1982**	$1.0 \cdot 10^{3}$
**Kunen et al., 1983**	$2.5 \cdot 10^{2}$
**Lind et al., 1987**	$8.0 \cdot 10^{2}$
**Hob et al., 1991**	$4.8 \cdot 10^{3}$
**Dovrou et al., 2019**	$1.6(\pm 0.23) \cdot 10^{3}$

Table S4. Rate constants of $SO_{2,aq}$ oxidation by peroxides. Rate constants of the examined reactions calculated by the second order solution model simulations[13]. The values corresponding to the reaction of $SO_{2,aq}$+H_2O_2 are the lower values of the rate constants.

		**Rate constant ($\mathbf{M^{-1}\ s^{-1}}$)**
**$SO_{2,aq}$+H_2O_2**	pH=5.5	$1.60(\pm 0.23) \cdot 10^3$
**$SO_{2,aq}$+1, 2-ISOPOOH**	pH=5.5	$1.65(\pm 0.28) \cdot 10^3$
	pH=4.5	$1.00(\pm 0.28) \cdot 10^3$
	pH=3.0	$1.00(\pm 0.42) \cdot 10^3$
**$SO_{2,aq}$+4, 3-ISOPOOH**	pH=5.5	$1.80(\pm 0.23) \cdot 10^2$
	pH=4.5	$0.90(\pm 0.18) \cdot 10^2$
	pH=3.0	$2.90(\pm 0.30) \cdot 10^2$
**$SO_{2,aq}$+HMHP**	pH=5.5	$8.00(\pm 1.20) \cdot 10^3$
	pH=4.5	$1.50(\pm 0.71) \cdot 10^4$
	pH=3.0	$1.40(\pm 0.79) \cdot 10^4$

Table S5. Percent contribution pathways in the production of sulfate considering fast HMHP equilibrium obtained by GEOS-Chem at altitude of 0-10 km.

	% Contribution										
	**Gas-phase OH**	**Gas-phase Criegees**	**H_2O_2**	**O_3**	**O_2 (metal)**	**Sea salt aerosol**	**HOBr**	**ISOPOOH**	**Direct HMHP**	**HCHO-catalysis**	**HMS**
**Global contribution Current conditions**	21.3%	0.0%	37.8%	12.6%	0.9%	4.5%	4.3%	0.8%	0.2%	17.3%	0.2%
**Global contribution Pre-industrial conditions**	13.8%	0.0%	41.5%	10.1%	0.2%	9.1%	1.4%	0.5%	0.1%	23.2%	0.2%
**Amazon contribution Current conditions**	4.9%	0.2%	10.3%	4.5%	0.0%	0.0%	0.9%	21.9%	0.4%	56.7%	0.1%
**SE-US contribution Current conditions**	32.1%	0.3%	36.1%	11.2%	0.0%	0.0%	0.7%	2.8%	0.1%	16.7%	0.0%
**Congo Basin contribution Current conditions**	14.3%	0.4%	17.5%	4.0%	0.4%	0.0%	1.0%	8.7%	0.5%	53.1%	0.1%
**East China contribution Current conditions**	62.8%	0.1%	17.4%	9.4%	8.2%	0.0%	0.5%	0.1%	0.1%	1.4%	0.0%
**India contribution Current conditions**	47.0%	0.1%	15.2%	27.2%	1.1%	0.7%	1.1%	0.4%	0.6%	6.7%	0.0%
**Indonesia contribution Current conditions**	15.47%	0.02%	51.55%	7.95%	0.01%	1.22%	1.54%	3.3%	0.1%	18.9%	0.0%

Table S5 (Continued)

	% Contribution										
	Gas-phase OH	Gas-phase Criegees	H_2O_2	O_3	O_2 (metal)	Sea salt aerosol	HOBr	ISOPOOH	Direct HMHP	HCHO-catalysis	HMS
**Amazon contribution Pre-industrial conditions**	11.9%	0.5%	9.7%	4.7%	0.0%	0.0%	0.5%	12.4%	0.3%	59.6%	0.2%
**SE-US contribution Pre-industrial conditions**	5.6%	0.1%	33.2%	1.4%	0.0%	0.0%	1.5%	2.2%	0.0%	56.0%	0.1%
**Congo Basin contribution Pre-industrial conditions**	20.3%	0.5%	24.4%	1.7%	0.4%	0.0%	0.4%	3.7%	0.4%	48.1%	0.1%
**East China contribution Pre-industrial conditions**	11.1%	0.0%	43.2%	3.2%	0.1%	1.5%	0.9%	2.7%	0.0%	37.1%	0.3%
**India contribution Pre-industrial conditions**	14.5%	0.0%	12.9%	17.3%	0.8%	18.4%	2.0%	5.7%	1.7%	26.5%	0.2%
**Indonesia contribution Pre-industrial conditions**	9.4%	0.0%	65.1%	3.1%	0.0%	3.7%	0.7%	2.0%	0.0%	16.0%	0.0%

Table S6. Percent contribution pathways in the production of sulfate considering medium HMHP equilibrium obtained by GEOS-Chem at altitude of 0-10 km.

	% Contribution										
	**Gas-phase OH**	**Gas-phase Criegees**	**H_2O_2**	**O_3**	**O_2 (metal)**	**Sea salt aerosol**	**HOBr**	**ISOPOOH**	**Direct HMHP**	**HCHO-catalysis**	**HMS**
**Global contribution Current conditions**	22.1%	0.0%	47.3%	13.4%	0.9%	4.7%	4.4%	0.9%	1.1%	4.9%	0.2%
**Global contribution Pre-industrial conditions**	14.6%	0.0%	55.0%	11.0%	0.2%	9.7%	1.4%	0.5%	0.5%	6.9%	0.2%
**Amazon contribution Current conditions**	5.0%	0.2%	16.0%	4.5%	0.0%	0.0%	0.9%	22.5%	14.5%	36.2%	0.1%
**SE-US contribution Current conditions**	32.8%	0.3%	40.3%	11.7%	0.0%	0.0%	0.6%	3.1%	4.7%	6.3%	0.0%
**Congo Basin contribution Current conditions**	14.7%	0.4%	24.2%	4.2%	0.4%	0.0%	1.0%	8.8%	11.6%	34.6%	0.1%
**East China contribution Current conditions**	62.7%	0.1%	17.8%	9.4%	8.1%	0.0%	0.5%	0.1%	0.6%	0.6%	0.0%
**India contribution Current conditions**	47.4%	0.1%	18.3%	27.7%	1.1%	0.7%	1.1%	0.4%	1.7%	1.5%	0.0%
**Indonesia contribution Current conditions**	16.1%	0.0%	57.6%	8.6%	0.0%	1.3%	1.6%	3.6%	3.3%	7.8%	0.1%

Table S6 (Continued)

	% Contribution										
	**Gas-phase OH**	**Gas-phase Criegees**	**H_2O_2**	**O_3**	**O_2 (metal)**	**Sea salt aerosol**	**HOBr**	**ISOPOOH**	**Direct HMHP**	**HCHO-catalysis**	**HMS**
**Amazon contribution Pre-industrial conditions**	12.2%	0.6%	15.9%	4.7%	0.0%	0.0%	0.5%	12.8%	9.6%	43.4%	0.3%
**SE-US contribution Pre-industrial conditions**	6.1%	0.1%	47.1%	1.6%	0.0%	0.0%	1.5%	2.8%	1.1%	39.5%	0.1%
**Congo Basin contribution Pre-industrial conditions**	20.8%	0.5%	29.1%	1.7%	0.4%	0.0%	0.4%	3.9%	6.6%	36.4%	0.1%
**East China contribution Pre-industrial conditions**	11.7%	0.0%	54.0%	3.5%	0.1%	1.6%	0.9%	3.2%	1.2%	23.6%	0.3%
**India contribution Pre-industrial conditions**	15.2%	0.0%	17.9%	17.0%	0.7%	19.1%	2.0%	5.9%	7.5%	14.5%	0.2%
**Indonesia contribution Pre-industrial conditions**	9.6%	0.0%	69.7%	3.3%	0.0%	3.9%	0.7%	2.2%	0.7%	9.8%	0.0%

Table S7. Percent contribution pathways in the production of sulfate considering slow HMHP equilibrium obtained by GEOS-Chem at altitude of 0-10 km.

	% Contribution										
	**Gas-phase OH**	**Gas-phase Criegees**	**H_2O_2**	**O_3**	**O_2 (metal)**	**Sea salt aerosol**	**HOBr**	**ISOPOOH**	**Direct HMHP**	**HCHO-catalysis**	**HMS**
**Global contribution Current conditions**	22.3%	0.0%	49.9%	13.8%	1.0%	4.8%	4.4%	1.0%	2.2%	0.4%	0.2%
**Global contribution Pre-industrial conditions**	14.9%	0.0%	59.1%	11.4%	0.2%	9.8%	1.4%	0.6%	1.7%	0.6%	0.2%
**Amazon contribution Current conditions**	5.9%	0.3%	22.8%	6.0%	0.0%	0.0%	1.0%	29.7%	32.3%	1.8%	0.2%
**SE-US contribution Current conditions**	33.3%	0.3%	42.6%	12.0%	0.0%	0.0%	0.6%	3.4%	7.8%	0.0%	0.1%
**Congo Basin contribution Current conditions**	16.3%	0.5%	32.8%	5.5%	0.5%	0.0%	1.1%	12.5%	27.8%	3.0%	0.1%
**East China contribution Current conditions**	62.7%	0.1%	18.0%	9.4%	8.1%	0.0%	0.5%	0.1%	0.9%	0.0%	0.0%
**India contribution Current conditions**	47.5%	0.1%	18.5%	27.9%	1.1%	0.7%	1.1%	0.4%	2.0%	0.7%	0.0%
**Indonesia contribution Current conditions**	16.4%	0.0%	60.5%	8.9%	0.0%	1.3%	1.6%	4.1%	6.9%	0.1%	0.1%

Table S7 (Continued)

	% Contribution										
	Gas-phase OH	Gas-phase Criegees	H_2O_2	O_3	O_2 (metal)	Sea salt aerosol	HOBr	ISOPOOH	Direct HMHP	HCHO-catalysis	HMS
**Amazon contribution Pre-industrial conditions**	13.8%	0.6%	23.3%	5.9%	0.0%	0.0%	0.6%	17.5%	31.2%	6.8%	0.3%
**SE-US contribution Pre-industrial conditions**	6.7%	0.1%	66.1%	2.0%	0.0%	0.0%	1.6%	3.8%	15.6%	4.0%	0.1%
**Congo Basin contribution Pre-industrial conditions**	23.0%	0.6%	38.9%	2.2%	0.5%	0.0%	0.4%	5.4%	23.5%	5.4%	0.1%
**East China contribution Pre-industrial conditions**	12.5%	0.0%	65.8%	3.8%	0.1%	1.7%	0.9%	3.9%	9.2%	1.8%	0.4%
**India contribution Pre-industrial conditions**	15.9%	0.0%	20.3%	18.3%	0.8%	19.7%	2.1%	6.5%	12.6%	3.5%	0.3%
**Indonesia contribution Pre-industrial conditions**	9.9%	0.0%	74.3%	3.4%	0.0%	4.0%	0.7%	2.4%	4.6%	0.5%	0.1%

Table S8. Ratio of data considering fast HMHP equilibrium to the base case of the simulations of GEOS-Chem. Values lower than 100 indicate decrease of the examined parameter when the HMHP oxidation pathway is implemented in the simulations.

	Ratio of data of equilibrium examined to base case (%)			
	**SO_2 burden**	**SO_4^{2-} burden**	**SO_4^{2-} production per year**	**SO_2 lifetime**
**Global contribution Current conditions**	97.6%	102.9%	103.8%	94.8%
**Global contribution Pre-industrial conditions**	96.8%	103.8%	105.0%	93.4%
**Amazon contribution Current conditions**	77.9%	124.5%	190.8%	52.9%
**SE-US contribution Current conditions**	96.6%	108.9%	109.0%	90.5%
**Congo Basin contribution Current conditions**	81.1%	113.6%	137.7%	66.2%
**East China contribution Current conditions**	99.9%	100.4%	100.4%	99.6%
**India contribution Current conditions**	99.4%	101.8%	101.9%	98.6%
**Indonesia contribution Current conditions**	94.4%	108.9%	110.9%	87.0%
**Amazon contribution Pre-industrial conditions**	79.3%	122.0%	177.3%	55.1%
**SE-US contribution Pre-industrial conditions**	90.6%	132.1%	132.4%	76.3%
**Congo Basin contribution Pre-industrial conditions**	83.6%	111.2%	129.8%	70.0%
**East China contribution Pre-industrial conditions**	98.1%	112.2%	110.0%	91.2%
**India contribution Pre-industrial conditions**	97.3%	108.7%	109.2%	94.2%
**Indonesia contribution Pre-industrial conditions**	95.4%	107.1%	108.7%	89.2%

Tables S8 (Continued)

	Ratio of data of equilibrium examined to base case (%)			
	**SO_2 burden**	**SO_4^{2-} burden**	**SO_4^{2-} production per year**	**SO_2 lifetime**
**Northern hemisphere summer contribution Current conditions**	96.1%	103.5%	104.3%	93.3%
**Northern hemisphere summer contribution Pre-industrial conditions**	95.2%	104.5%	105.5%	91.7%
**Northern hemisphere summer Amazon contribution Current conditions**	82.6%	123.4%	172.7%	56.7%
**Northern hemisphere summer SE-US contribution Current conditions**	90.5%	121.7%	121.6%	77.6%
**Northern hemisphere summer Congo Basin contribution Current conditions**	82.8%	111.0%	126.6%	68.5%
**Northern hemisphere summer East China contribution Current conditions**	99.8%	100.8%	100.5%	99.4%
**Northern hemisphere summer India contribution Current conditions**	97.8%	104.5%	103.4%	96.7%
**Northern hemisphere summer Indonesia contribution Current conditions**	94.8%	109.0%	111.2%	87.2%
**Northern hemisphere summer Amazon contribution Pre-industrial conditions**	84.8%	119.1%	155.4%	60.8%
**Northern hemisphere summer SE-US contribution Pre-industrial conditions**	82.9%	154.6%	154.3%	63.7%
**Northern hemisphere summer Congo Basin contribution Pre-industrial conditions**	85.2%	109.1%	121.4%	72.2%
**Northern hemisphere summer East China contribution Pre-industrial conditions**	95.5%	123.9%	112.7%	87.9%
**Northern hemisphere summer India contribution Pre-industrial conditions**	93.1%	116.4%	112.0%	89.9%
**Northern hemisphere summer Indonesia contribution Pre-industrial conditions**	95.6%	107.4%	109.1%	89.1%

Table S9. Ratio of data considering medium HMHP equilibrium to the base case of the simulations of GEOS-Chem. Values lower than 100 indicate decrease of the examined parameter when the HMHP oxidation pathway is implemented in the simulations.

	**Ratio of data of equilibrium examined to base case (%)**			
	**SO_2 burden**	**SO_4^{2-} burden**	**SO_4^{2-} production per year**	**SO_2 lifetime**
**Global contribution Current conditions**	98.9%	101.6%	101.9%	97.7%
**Global contribution Pre-industrial conditions**	99.5%	101.9%	102.5%	98.2%
**Amazon contribution Current conditions**	79.0%	122.4%	189.5%	54.0%
**SE-US contribution Current conditions**	97.5%	106.6%	107.6%	92.2%
**Congo Basin contribution Current conditions**	83.7%	111.9%	137.5%	68.4%
**East China contribution Current conditions**	99.9%	100.4%	100.4%	99.5%
**India contribution Current conditions**	99.6%	101.3%	101.4%	99.3%
**Indonesia contribution Current conditions**	96.3%	106.0%	108.3%	90.5%
**Amazon contribution Pre-industrial conditions**	81.7%	119.5%	176.4%	57.0%
**SE-US contribution Pre-industrial conditions**	96.8%	128.8%	131.0%	82.3%
**Congo Basin contribution Pre-industrial conditions**	86.4%	109.2%	129.3%	72.6%
**East China contribution Pre-industrial conditions**	102.2%	110.0%	108.1%	96.7%
**India contribution Pre-industrial conditions**	99.9%	107.5%	106.9%	99.3%
**Indonesia contribution Pre-industrial conditions**	96.9%	104.8%	107.4%	91.6%

Table S9 (Continued)

	Ratio of data of equilibrium examined to base case (%)			
	SO_2 burden	SO_4^{2-} burden	SO_4^{2-} production per year	SO_2 lifetime
**Northern hemisphere summer contribution Current conditions**	98.0%	102.2%	102.3%	96.7%
**Northern hemisphere summer contribution Pre-industrial conditions**	97.9%	102.6%	102.9%	96.5%
**Northern hemisphere summer Amazon contribution Current conditions**	84.0%	120.6%	170.5%	58.4%
**Northern hemisphere summer SE-US contribution Current conditions**	92.6%	117.0%	118.5%	81.0%
**Northern hemisphere summer Congo Basin contribution Current conditions**	86.4%	108.8%	125.2%	72.1%
**Northern hemisphere summer East China contribution Current conditions**	99.9%	100.6%	100.5%	99.4%
**Northern hemisphere summer India contribution Current conditions**	98.2%	103.9%	102.7%	97.6%
**Northern hemisphere summer Indonesia contribution Current conditions**	96.4%	106.3%	108.5%	90.6%
**Northern hemisphere summer Amazon contribution Pre-industrial conditions**	87.0%	115.9%	153.5%	62.9%
**Northern hemisphere summer SE-US contribution Pre-industrial conditions**	88.9%	151.2%	152.9%	68.9%
**Northern hemisphere summer Congo Basin contribution Pre-industrial conditions**	88.4%	107.0%	119.9%	75.8%
**Northern hemisphere summer East China contribution Pre-industrial conditions**	100.5%	120.8%	109.4%	95.3%
**Northern hemisphere summer India contribution Pre-industrial conditions**	93.3%	116.6%	111.6%	91.5%
**Northern hemisphere summer Indonesia contribution Pre-industrial conditions**	96.9%	105.4%	107.8%	91.5%

Table S10. Ratio of data considering slow HMHP equilibrium to the base case of the simulations of GEOS-Chem. Values lower than 100 indicate decrease of the examined parameter when the HMHP oxidation pathway is implemented in the simulations.

	**Ratio of data of equilibrium examined to base case (%)**			
	**SO_2 burden**	**SO_4^{2-} burden**	**SO_4^{2-} production per year**	**SO_2 lifetime**
**Global contribution Current conditions**	99.4%	101.1%	101.3%	98.7%
**Global contribution Pre-industrial conditions**	100.7%	101.1%	101.6%	100.1%
**Amazon contribution Current conditions**	84.8%	116.9%	171.5%	62.7%
**SE-US contribution Current conditions**	98.0%	105.4%	106.5%	93.4%
**Congo Basin contribution Current conditions**	89.1%	108.6%	128.8%	76.6%
**East China contribution Current conditions**	99.9%	100.3%	100.5%	99.5%
**India contribution Current conditions**	99.7%	101.1%	101.2%	99.4%
**Indonesia contribution Current conditions**	97.2%	104.7%	106.6%	92.6%
**Amazon contribution Pre-industrial conditions**	87.7%	115.0%	164.2%	64.8%
**SE-US contribution Pre-industrial conditions**	101.8%	126.4%	124.7%	90.8%
**Congo Basin contribution Pre-industrial conditions**	91.9%	105.6%	122.3%	81.2%
**East China contribution Pre-industrial conditions**	105.5%	108.4%	104.1%	103.4%
**India contribution Pre-industrial conditions**	102.7%	105.8%	104.3%	103.5%
**Indonesia contribution Pre-industrial conditions**	97.9%	103.2%	105.1%	94.5%

Table S10 (Continued)

	Ratio of data of equilibrium examined to base case (%)			
	SO_2 burden	SO_4^{2-} burden	SO_4^{2-} production per year	SO_2 lifetime
**Northern hemisphere summer contribution Current conditions**	98.7%	101.6%	101.6%	97.9%
**Northern hemisphere summer contribution Pre-industrial conditions**	99.2%	101.6%	101.9%	98.7%
**Northern hemisphere summer Amazon contribution Current conditions**	89.0%	114.8%	154.9%	67.0%
**Northern hemisphere summer SE-US contribution Current conditions**	94.1%	113.7%	115.8%	83.9%
**Northern hemisphere summer Congo Basin contribution Current conditions**	91.7%	105.7%	118.4%	80.5%
**Northern hemisphere summer East China contribution Current conditions**	99.9%	100.5%	100.5%	99.4%
**Northern hemisphere summer India contribution Current conditions**	98.4%	103.4%	102.3%	97.9%
**Northern hemisphere summer Indonesia contribution Current conditions**	97.3%	105.0%	106.8%	92.6%
**Northern hemisphere summer Amazon contribution Pre-industrial conditions**	91.5%	110.4%	143.1%	69.9%
**Northern hemisphere summer SE-US contribution Pre-industrial conditions**	97.0%	147.3%	145.3%	78.9%
**Northern hemisphere summer Congo Basin contribution Pre-industrial conditions**	94.1%	103.7%	113.3%	85.3%
**Northern hemisphere summer East China contribution Pre-industrial conditions**	105.2%	118.1%	102.9%	105.7%
**Northern hemisphere summer India contribution Pre-industrial conditions**	96.6%	114.6%	109.2%	95.4%
**Northern hemisphere summer Indonesia contribution Pre-industrial conditions**	98.0%	103.8%	105.4%	94.5%
